# Dysregulation of the miR‐194–CUL4B negative feedback loop drives tumorigenesis in non‐small‐cell lung carcinoma

**DOI:** 10.1002/1878-0261.12038

**Published:** 2017-02-21

**Authors:** Jun Mi, Yongxin Zou, Xiaohua Lin, Juanjuan Lu, Xiaochen liu, Hui Zhao, Xiang Ye, Huili Hu, Baichun Jiang, Bo Han, Changshun Shao, Yaoqin Gong

**Affiliations:** ^1^ The Key Laboratory of Experimental Teratology Ministry of Education and Department of Molecular Medicine and Genetics Shandong University School of Basic Medical Sciences Jinan China; ^2^ Shandong Provincial Key Laboratory of Oral Tissue Regeneration Shandong University School of Stomatology Jinan China; ^3^ Department of Pathology Shandong University School of Basic Medical Sciences Jinan China; ^4^ Department of Pathology Shandong University Qilu Hospital Jinan China; ^5^ Department of Genetics/Human Genetics Institute of New Jersey Piscataway NJ USA

**Keywords:** CUL4B, double‐negative feedback loop, H2AK119 monoubiquitination, lung cancer, microRNA

## Abstract

Cullin 4B (CUL4B), a scaffold protein that assembles CRL4B ubiquitin ligase complexes, is overexpressed in many types of cancers and represses many tumor suppressors through epigenetic mechanisms. However, the mechanisms by which CUL4B is upregulated remain to be elucidated. Here, we show that CUL4B is upregulated in non‐small‐cell lung carcinoma (NSCLC) tissues and is critically required for cell proliferation and migration *in vitro* and for xenograft tumor formation *in vivo*. We found that microRNA‐194 (miR‐194) and CUL4B protein were inversely correlated in cancer specimens and demonstrated that miR‐194 could downregulate CUL4B by directly targeting its 3′‐UTR. We also showed that CUL4B could be negatively regulated by p53 in a miR‐194‐dependent manner. miR‐194 was further shown to attenuate the malignant phenotype of lung cancer cells by downregulating CUL4B. Interestingly, CRL4B also epigenetically represses miR‐194 by catalyzing monoubiquitination at H2AK119 and by coordinating with PRC2 to promote trimethylation at H3K27 at the gene clusters encoding miR‐194. RBX1, another component in CRL4B complex, is also targeted by miR‐194 in NSCLC cells. Our results thus establish a double‐negative feedback loop between miR‐194 and CRL4B, dysregulation of which contributes to tumorigenesis. The function of miR‐194 as a negative regulator of CUL4B has therapeutic implications in lung cancer.

**Abbreviations**
CRL4B: cullin 4B‐RING‐based E3 ubiquitin ligases
CUL4B: cullin 4B
HDAC3: histone deacetylase 3
NSCLC: non‐small‐cell lung carcinoma

## 1 Introduction

Cullin 4B (CUL4B) acts as a scaffold that assembles DDB1, RBX1 and substrate receptors to form cullin 4B‐RING‐based E3 ubiquitin ligases (CRL4B) (Petroski and Deshaies, 2005). Lack of CUL4B greatly compromises cell proliferation, extra embryonic development, hematopoiesis, neurogenesis, and spermatogenesis (Chen *et al*., 2012; Ji *et al*., 2014; Jiang *et al*., 2012; Liu *et al*., 2012; Nakagawa and Xiong, 2011; Qian *et al*., 2015; Tarpey *et al*., 2007; Vulto‐van Silfhout *et al*., 2015; Yin *et al*., 2016; Zhao *et al*., 2015; Zou *et al*., 2007, 2009). On the other hand, CUL4B is overexpressed in a variety of cancers and is crucial for the maintenance of the malignant behaviors of cancer cells (Hu *et al*., 2012; Jiang *et al*., 2013; Yuan *et al*., 2015). Mechanistically, CRL4B can catalyze either polyubiquitination for proteasomal degradation or monoubiquitination at H2A for epigenetic modification (He *et al*., 2013; Hu *et al*., 2012; Li *et al*., 2011; Wei *et al*., 2015; Zou *et al*., 2009). We recently showed that CRL4B is capable of repressing a number of tumor suppressors including p16, PTEN, Wnt antagonists, and IGFBP3 through catalyzing H2AK119 monoubiquitination (H2AK119ub1) at their promoters (Hu *et al*., 2012; Yang *et al*., 2015; Yuan *et al*., 2015). While CUL4B was demonstrated to possess oncogenic properties in those studies, the mechanisms by which CUL4B is upregulated remain to be elucidated.

Noncoding RNAs, especially microRNAs (miRNAs), can negatively regulate their target genes by binding to the 3′‐UTR of mRNAs, thus inhibiting protein synthesis (Bartel, 2004). Many tumor suppressors and oncogenes have been identified to be targets of miRNAs (Calin and Croce, 2006; Kong *et al*., 2012), and aberrant miRNAs expression observed in cancer often has pathogenic and prognostic implications (Kong *et al*., 2012). Recent studies showed that miRNAs themselves are subjected to epigenetic regulation. For example, miR‐886, which targets PLK1 and TGF‐β1, was reported to be silenced by DNA hypermethylation in lung cancer (Cao *et al*., 2013), and miR‐29 was found to be epigenetically repressed by MYC through a corepressor complex with histone deacetylase 3 (HDAC3) and enhancer of zeste homolog 2 (EZH2) in aggressive B‐cell lymphomas (Zhang *et al*., 2012). However, the mechanisms underlying the expressional regulations of a large number of miRNAs remain to be characterized.

In this study, we investigated the role of CUL4B in lung cancer. We found that CUL4B was upregulated in lung cancer tissues and contributes to proliferation, migration, and invasion of non‐small‐cell lung cancer (NSCLC) cells. We further showed that CUL4B was a direct target of miR‐194. Interestingly, CRL4B also represses miR‐194 by catalyzing monoubiquitination at H2AK119 at the miR‐194 promoter. Therefore, CRL4B and miR‐194 effectively form a double‐negative feedback loop in tumorigenesis.

## 2 Materials and methods

### 2.1 Cell culture and manipulation

All cells were obtained from ATCC. 293T (CRL‐3216) cells were cultured in DMEM (Gibco, Invitrogen, Grand Island, NY, USA). H1299 (CRL‐5803) and A549 (CCL‐185™) cells were cultured in RPMI 1640 (Gibco). All media were supplemented with 10% FBS. Cells were maintained at 37 °C with 5% CO_2_. Plasmids, siRNA transfections and stable knockdown of CUL4B were performed as previously described (Wei *et al*., 2015; Zou *et al*., 2013).

### 2.2 Cell proliferation and colony formation assays

Cell proliferation was measured by MTT assay as previously described (Zou *et al*., 2013). The colony formation assay was performed as previously described (Yuan *et al*., 2015).

### 2.3 Wound‐healing and transwell migration assays

For wound‐healing assay, the wound was generated by scratching with a 200‐μL pipette tip. The horizontal distance from the initial wound was measured, and the rate of wound closure was calculated as follows: [(mean wound width‐mean remaining width)/mean wound width] × 100 (%). Experiments were carried out in triplicate, and three randomly selected fields of each well were recorded. The transwell migration assay was performed as previously described (Hu *et al*., 2012). All experiments were performed in triplicate, and results were expressed as the average number of cells per field.

### 2.4 EdU incorporation and flow cytometry assays

EdU (5‐ethynyl‐2′‐deoxyuridine) incorporation was detected using the Cell‐Light™ EdU DNA Cell Proliferation Kit (Ribobio, Guangzhou, China). The cell cycle distribution was performed as previously described (Zou *et al*., 2013).

### 2.5 Tumor xenograft assay

5 × 10^6^ cells were injected into the dorsal flank of female 5‐ to 6‐week‐old BALB/c nude mice. Tumor volumes were measured by measuring the longest diameter (*L*) and the shortest diameter (*S*) with calipers and calculated with the formula: (*L* × *S* ^2^) × 0.5. All experiments were performed in compliance with national regulations and approved by the Animal Care and Use Committee, Shandong University, School of Medicine.

### 2.6 Tissue specimens and immunohistochemistry

This study was approved by Medical Ethics Committee, Shandong University School of Medicine. Thirty lung cancer and paired adjacent tissue samples were obtained from surgically removed tissues. Informed consent was obtained from each patient. The tissue microarrays were purchased from Superchip (Shanghai, China, Cat: HLug‐Ade150Sur‐02 and HLug‐Ade050CD‐01). Immunohistochemical staining was performed as previously reported (Yuan *et al*., 2015). The CUL4B expressions were quantified using a four‐value score for intensity (0 = negative, 1 = light, 2 = moderate, and 3 = intense) and percentage of the extent of reactivity (0 = <10%, 1 = 10–29%, 2 = 30–59%, and 3 > 60% positive cells). An immunohistochemical expression score was obtained by multiplying the intensity and reactivity extension values (range, 0–9).

### 2.7 RNA extraction and reverse transcription PCR

Extraction of total RNA, reverse transcription PCR, and real‐time quantitative PCR (qPCR) assay was performed as described previously (Zou *et al*., 2013). Primer sequences used for qPCR are listed in Table S1. All primers used for miRNA qPCR were purchased from GeneCopoeia (GeneCopoeia, Inc., Rockville, MD, USA). High‐throughput cancer‐related miRNA qPCR analyses were performed using miProfile™ PAM‐HC96 Human Cancer miRNA qPCR Array (GeneCopoeia).

### 2.8 Western blot and antibodies

Western blot analysis was performed as described previously (Zou *et al*., 2013). The primary antibodies are listed in Table S2.

### 2.9 Plasmids and luciferase assays

pCMV‐Tag2B‐CUL4B that expresses flag‐tagged wild‐type CUL4B was described previously (Zou *et al*., 2013). The pCMV‐Tag2B‐CUL4B (NLS‐del) was generated by subcloning the fragment from pEGFP‐C1‐CUL4BΔ37‐40 (Zou *et al*., 2009) in‐frame into the pCMV‐Tag 2B vector. The segments of CUL4B, RBX1, and N‐cadherin 3′‐UTR were cloned into the pmir‐GLO vector (Promega, Madison, WI, USA). The pmir‐GLO‐CUL4B 3′‐UTR vector containing mutated miR‐194 binding site was generated by site‐directed mutagenesis using overlap extension PCR. The pmir‐GLO‐RBX1 and the pmir‐GLO‐N‐cadherin 3′‐UTR vectors containing mutated miR‐194 binding site were generated by Fast Site‐Directed Mutagenesis Kit (TIANGEN, Beijing, China). Primer sequences are listed in Table S3. The 3′‐UTR reporter luciferase assays were performed as previously described (Zou *et al*., 2013).

### 2.10 ChIP assay

Chromatin immunoprecipitations (ChIPs) were performed as described previously (Hu *et al*., 2012). Primers and antibodies are listed in Tables S4 and S5.

### 2.11 Biotin‐miRNA pulldown

The biotinylated‐miRNA pulldown assay was adapted from Wani and Cloonan (2014). Briefly, 293T cells were transfected with 40 nm biotin‐control RNA or biotin‐miR‐194 mimic oligonucleotides (Ribobio). Twenty‐four hours later, cells were harvested in lysis buffer (10 mm KCl, 1.5 mm MgCl_2_, 10 mm Tris/Cl pH 7.5, 5 mm DTT, 0.5% Sigma‐IGEPAL CA‐630) supplemented with RNAsin (Promega) and protease inhibitor cocktail (Roche Applied Science, Indianapolis, IN, USA) and incubated on dry ice for 5 min. The cells were allowed to thaw out, and the lysate was isolated by centrifugation at 10 000× ***g*** for 15 min. The clear lysate was added with NaCl to a final concentration of 1M. MyOne Streptavidin C1 Dynabeads (Invitrogen, Grand Island, NY, USA) were blocked for 2 h at room temperature in RNase/DNase‐free water containing 1 μg·μL^−1^ BSA and 1 μg·μL^−1^ yeast tRNA and then washed twice with wash buffer (lysis buffer plus 1 m NaCl). Cytoplasmic lysate was added to the beads and incubated for 2.5 h at room temperature. Beads were then washed three times with wash buffer. RNA bound to the beads as well as 1% of the extract (input RNA) was isolated using RNeasy kit (Qiagen, Hilden, Germany). Pulled‐down mRNA was quantified by qRT‐PCR. Values were normalized to GAPDH in the same sample and then to input (cellular RNA without incubation with beads). The primer for CUL4B 3′‐UTR detection is as follows: forward: 5′‐AGACCAAAATGAACGTGTTT‐3′; reverse: 5′‐ GAAGAGTTGGGATGCTTCTA‐3′.

### 2.12 Statistical analysis

Data were analyzed using spss 13.0 (SPSS Inc., Chicago, IL, USA). Unless otherwise stated, differences between the mean values were analyzed for significance using the two‐tailed unpaired *t*‐test. Correlation significance was assessed using chi‐square test and Pearson's correlation coefficient test. *P* ≤ 0.05 was considered to be statistically significant.

## 3 Results

### 3.1 CUL4B is overexpressed in NSCLC

**Figure 1:**
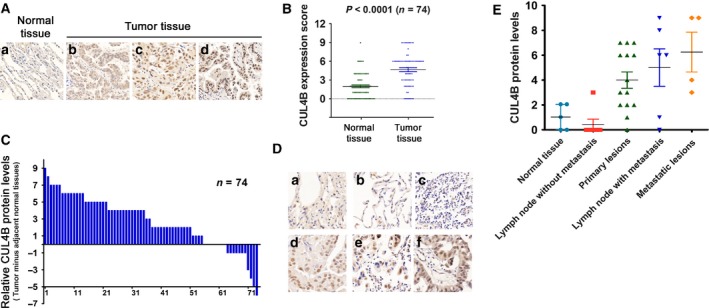
Cullin 4B protein is overexpressed in human NSCLC tissues. (A) Representative IHC straining of CUL4B in NSCLC tissues and the adjacent normal tissues. Staining intensity was classified using a 0‐to‐3 scale, corresponding to a, b, c, and d (a: 0, negative; b: 1, weak; c: 2, moderate; d: 3, strong). (B) Distribution of CUL4B IHC expression scores in the NSCLC and adjacent lung tissues. Error bars represent SEM;* P* values were determined by two‐tailed paired *t*‐test. (C) Relative CUL4B IHC scores in NSCLC and paired normal tissues. (D) Representative IHC straining of CUL4B in indicated tissues (a: normal lung; b: normal lung adjacent to tumor; c: lymph nodes without metastasis; d: primary lung tumor; e: metastatic lymph nodes; f: distant metastatic lesions). (E) Distribution of CUL4B expression levels in normal lung and primary or metastatic tissues in patients with NSCLC. Error bars represent SEM.

We examined CUL4B protein levels in 74 paired cases of lung adenocarcinoma specimens by immunohistochemistry. CUL4B was positively stained in >90% of samples and was mainly localized in the nuclei of cancer cells (Fig. 1A). CUL4B levels in NSCLC samples were significantly higher than in normal lung tissues (Fig. 1B). Moreover, CUL4B levels were higher in 55 (74.32%) lung adenocarcinoma samples than in matched noncancerous tissues (Fig. 1C).

We next analyzed the association between CUL4B expression and clinicopathological parameters. As shown in Table S6, expression levels of CUL4B were significantly correlated with tumor size (*P *=* *0.019). However, no statistically significant correlations were observed between expression of CUL4B and other clinicopathological parameters (*P* > 0.05).

We further examined CUL4B expression in normal lung, primary adenocarcinomas, nearby lymph nodes with or without metastasis, and distant metastatic lesions (Fig. 1D). Significant upregulation of CUL4B was observed in primary tumors, metastatic lymph nodes, and distant metastatic lesions. However, no significant difference in CUL4B expression was observed between primary tumors versus metastatic lymph nodes or distant metastatic lesions (Fig. 1E). Together, these results suggest that CUL4B is overexpressed in primary lung adenocarcinoma tissues and this increased expression is maintained in the metastatic tissues.

### 3.2 Knockdown of CUL4B inhibits the proliferation, migration, and invasion of NSCLC cells

**Figure 2:**
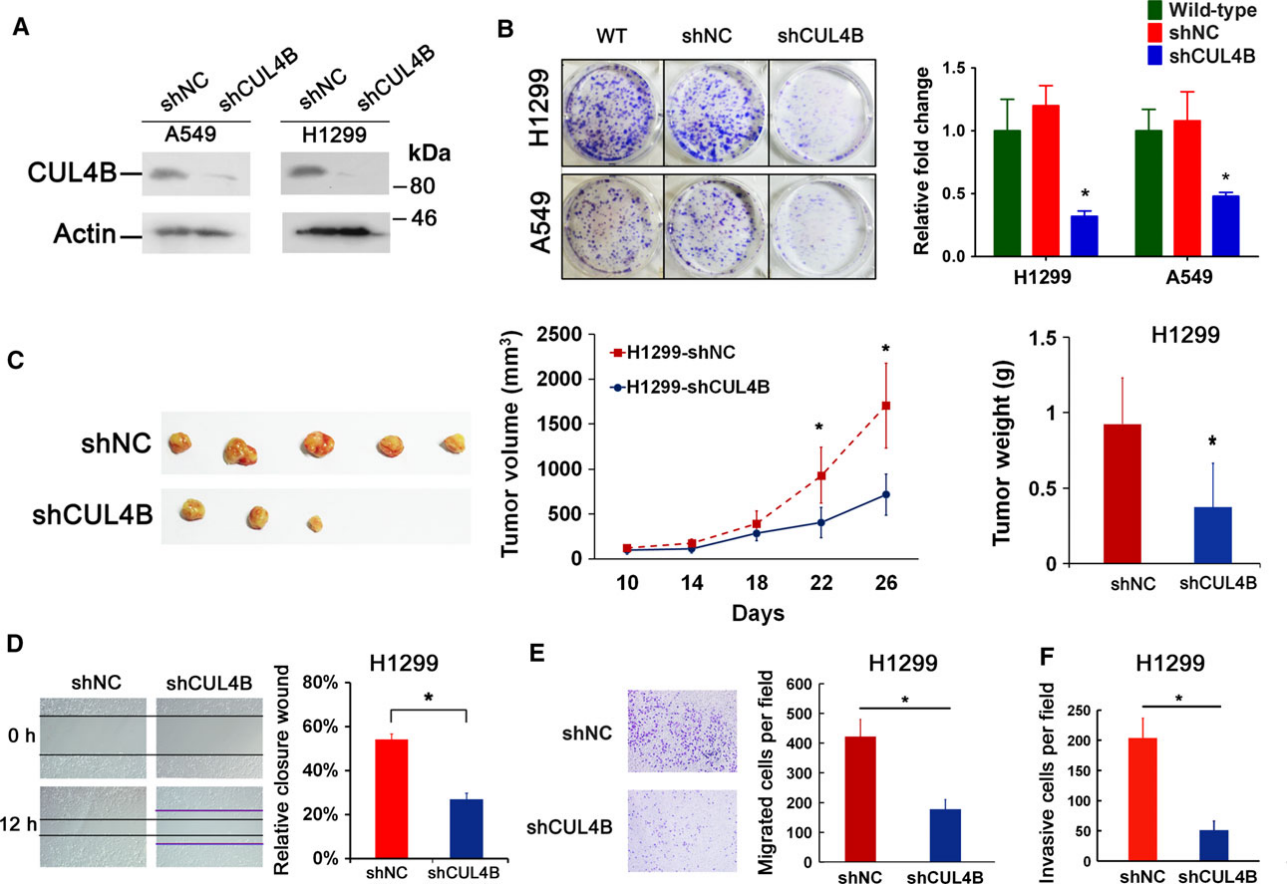
Knockdown of CUL4B inhibits proliferation, migration, and invasion of NSCLC cells. (A) Western blot showing knockdown efficiency of CUL4B in A549 and H1299 cells. (B) Colony formation efficiency of H1299 and A549 cells with CUL4B knockdown. Error bars represent SD. (C) Tumor formation by shCUL4B and control H1299 cells in nude mice, respectively. Shown are representative tumors 26 days after injection, and the tumor weight was measured. Error bars represent SD. (D–F) Migration and invasion ability of indicated cells was examined by wound‐healing assay (D), transwell migration assay (E), and Matrigel invasion assay (F). Error bars represent SD. Statistical comparisons were made using two‐tailed unpaired *t*‐test. *P < 0.05.

We next investigated the roles of CUL4B in cell proliferation, migration, and invasion. Clonogenic assay showed that CUL4B stable knockdown cells displayed much fewer and smaller colonies (Fig. 2A,B). MTT assays also showed significantly reduced growth in CUL4B knockdown cells (Fig. S1A,B). Consistently, shCUL4B cells showed a significant decrease in the percentage of EdU‐positive cells (Fig. S1C). A previously described siRNA (Zou *et al*., 2013) produced a similar effect (Fig. S1D,E). Furthermore, flow cytometry analysis demonstrated that CUL4B knockdown by siRNA increased the percentage of cells in S‐phase (Fig. S1F). These data suggest that knockdown of CUL4B may primarily inhibit cell cycle progression.

Then, we tested the roles of CUL4B in tumor growth *in vivo* in a xenograft model. While shNC cells produced tumors in all five mice, shCUL4B cells were less tumorigenic, producing tumors in three of five mice only at the end of observation (Fig. 2C, left). Tumors formed by shNC cells grew much faster than those by shCUL4B cells (Fig. 2C, middle and right). Moreover, the number of Ki67‐positive tumor cells in the shCUL4B group was significantly decreased compared with that in the shNC group (Fig. S1G). CUL4B knockdown in A549 cells similarly reduced their tumorigenicity *in vivo* (Fig. S1H). These results indicate that knockdown of CUL4B significantly inhibits tumor growth.

Wound‐healing and transwell assays showed that knockdown of CUL4B caused a significant decrease in cell migration (Fig. 2D,E). Furthermore, Matrigel invasion assay showed a great reduction in the number of invasive cells in the shCUL4B cells (Fig. 2F). Together, these results indicate that depletion of CUL4B can significantly inhibit the migration and invasion of NSCLC cells.

### 3.3 Identification of CUL4B as a target of miR‐194

**Figure 3:**
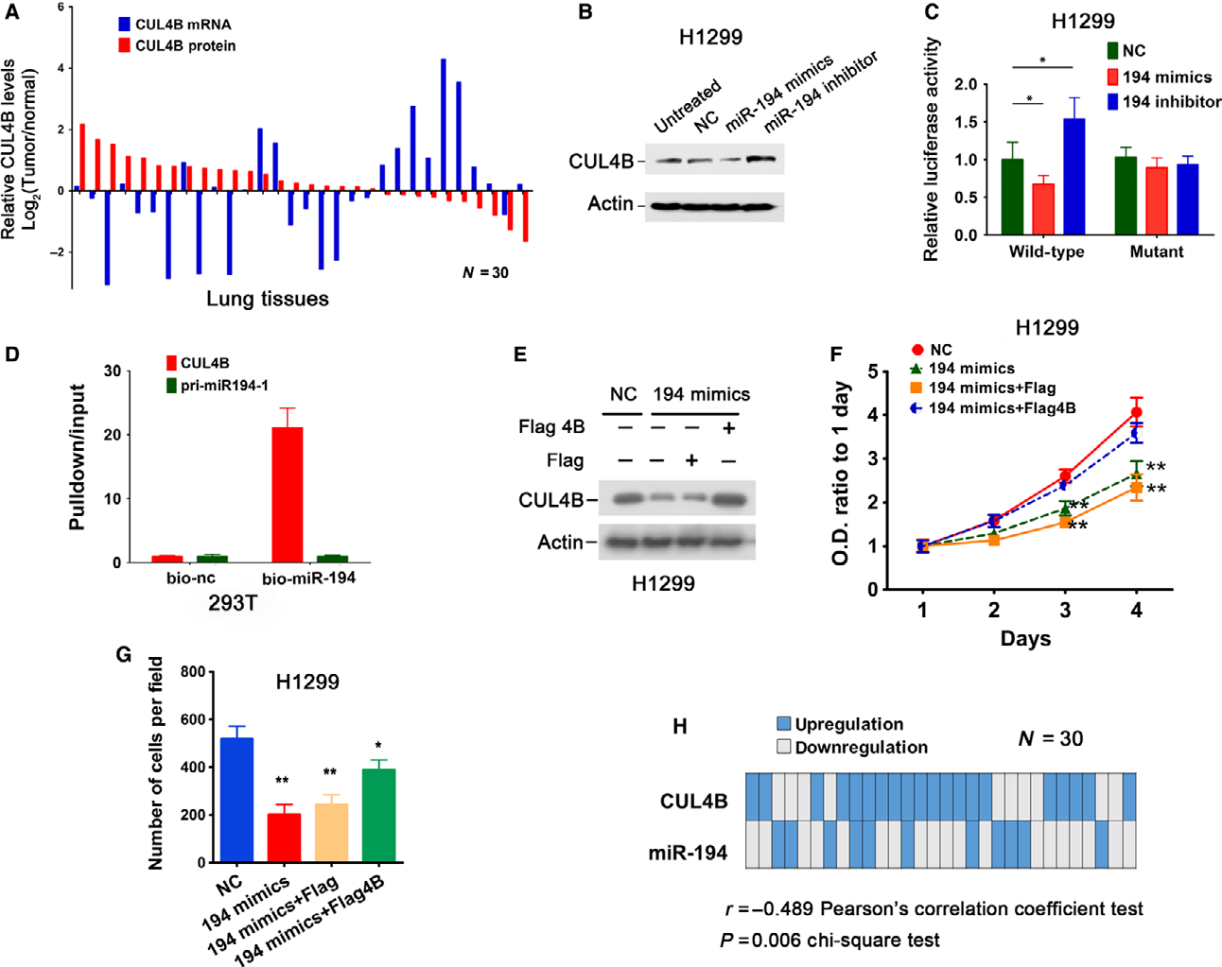
Cullin 4B is a target of miR‐194. (A) CUL4B expression levels in lung cancer tissues relative to those in paired adjacent nontumor tissues, as determined by western blot and real‐time PCR. (B) H1299 cells were transiently transfected with miR‐194 mimics, inhibitor, or negative control RNA (NC), respectively. Seventy‐two hours later, CUL4B protein levels were determined. (C) H1299 cells were transiently cotransfected with indicated RNA together with wild‐type or mutant pmir‐GLO‐CUL4B 3′‐UTR. Forty‐eight hours later, luciferase assay was performed. The luciferase activity in NC transfected cells was set as 1. Bars represent SD. *P < 0.05, two‐tailed unpaired *t*‐test. (D) 293T cells were transfected with 10 nm biotinylated miR‐194 mimics (bio‐194) or biotinylated control RNA (bio‐nc). Twenty‐four hours later, cells were harvested for pulldown analysis, followed by real‐time PCR detection of enriched CUL4B 3′‐UTR. Bars represent SEM of three independent experiments. (E–G) H1299 cells were transiently transfected with indicated RNA and flag‐tagged CUL4B (Flag 4B) or empty control plasmid pCMV‐Tag‐2B (Flag). Seventy‐two hours later, CUL4B protein levels were determined by western blot (E), proliferation of cells was examined by MTT assay (F), and migration ability of cells was examined by transwell migration assay (G). *P < 0.05; **P < 0.001 versus NC cells, two‐tailed unpaired *t*‐test. (H) Distribution of CUL4B protein and miR‐194 levels in 30 lung cancer samples; up‐ or downregulation is relative to adjacent nontumor tissues.

To gain insight into the mechanism by which CUL4B is upregulated in lung cancers, we examined the mRNA and protein levels of CUL4B in 30 paired specimens of lung cancer and adjacent normal tissues. Western blot showed that CUL4B protein was upregulated in 66.7% of tumor tissues when compared to adjacent normal tissues (Figs 3A and S2A). However, no corresponding changes in CUL4B mRNA levels were observed (Fig. 3A), suggesting that the upregulation of CUL4B in lung cancer may have occurred at posttranscriptional levels.

We next explored whether miRNAs are involved in the regulation of CUL4B. Three target prediction algorithms (TargetScan, miRanda, and miRDB) were utilized to predict the potential miRNAs that could target the 3′‐UTR of CUL4B. miR‐194 was predicted to target CUL4B by all three programs. We therefore transiently transfected mimics and inhibitors of miR‐194 into H1299 cells and examined CUL4B expression levels. As shown in Figs 3B and S2B,C, overexpression of miR‐194 significantly downregulated CUL4B at protein level, but not at mRNA level. Accordingly, transfection with anti‐miR‐194 inhibitors resulted in an increase in CUL4B proteins (Fig. 3B). Similar results were obtained with A549 cells (Fig. S2D).

The miR‐194 binding sites within the 3′‐UTR of CUL4B are highly conserved across different species (Fig. S2E). To determine whether miR‐194 acts directly on the 3′‐UTR of CUL4B, luciferase reporter vectors containing the 3′‐UTR of CUL4B with and without point mutations in the seed sequence of miR‐194 were cotransfected with mimics or inhibitors of miR‐194 or control miRNA into H1299 cells. Transfection with miR‐194 mimics dramatically reduced the luciferase activity of CUL4B 3′‐UTR, whereas transfection with miR‐194 inhibitor significantly increased the luciferase activity (Fig. 3C). In contrast, miR‐194 mimics and inhibitors had no effect on the luciferase activity of the reporter in which miR‐194 binding site was mutated (Fig. 3C). A biotin‐miR‐194 pulldown assay showed that CUL4B, but not control, mRNA was significantly enriched (Fig. 3D). Collectively, these results indicate that CUL4B is a direct target of miR‐194.

Overexpression of miR‐194 in lung cancer cells was reported to inhibit cell proliferation, migration, and invasion (Wu *et al*., 2014; Zhu *et al*., 2016). We next tested whether the inhibitory effect of miR‐194 on NSCLC cells might be mediated by its repression of CUL4B. The results showed that cell proliferation and migration were significantly inhibited by transfection with miR‐194 mimics in H1299 cells (Fig. 3F,G). However, ectopic expression of exogenous CUL4B could partially rescue the suppressive effect of miR‐194 (Fig. 3E–G). Hence, CUL4B serves as one of the functional effectors of miR‐194 in NSCLC cells.

We then determined the levels of miR‐194 in 30 human lung cancer tissues and paired adjacent normal lung tissues using real‐time PCR. Downregulation of miR‐194 was observed in 63.3% (19/30) of tumor tissues (Fig. 3H). Importantly, miR‐194 levels were negatively correlated with CUL4B protein levels in these tumor tissues (Fig. 3H).

### 3.4 p53–miR‐194 axis negatively regulates CUL4B expression

**Figure 4:**
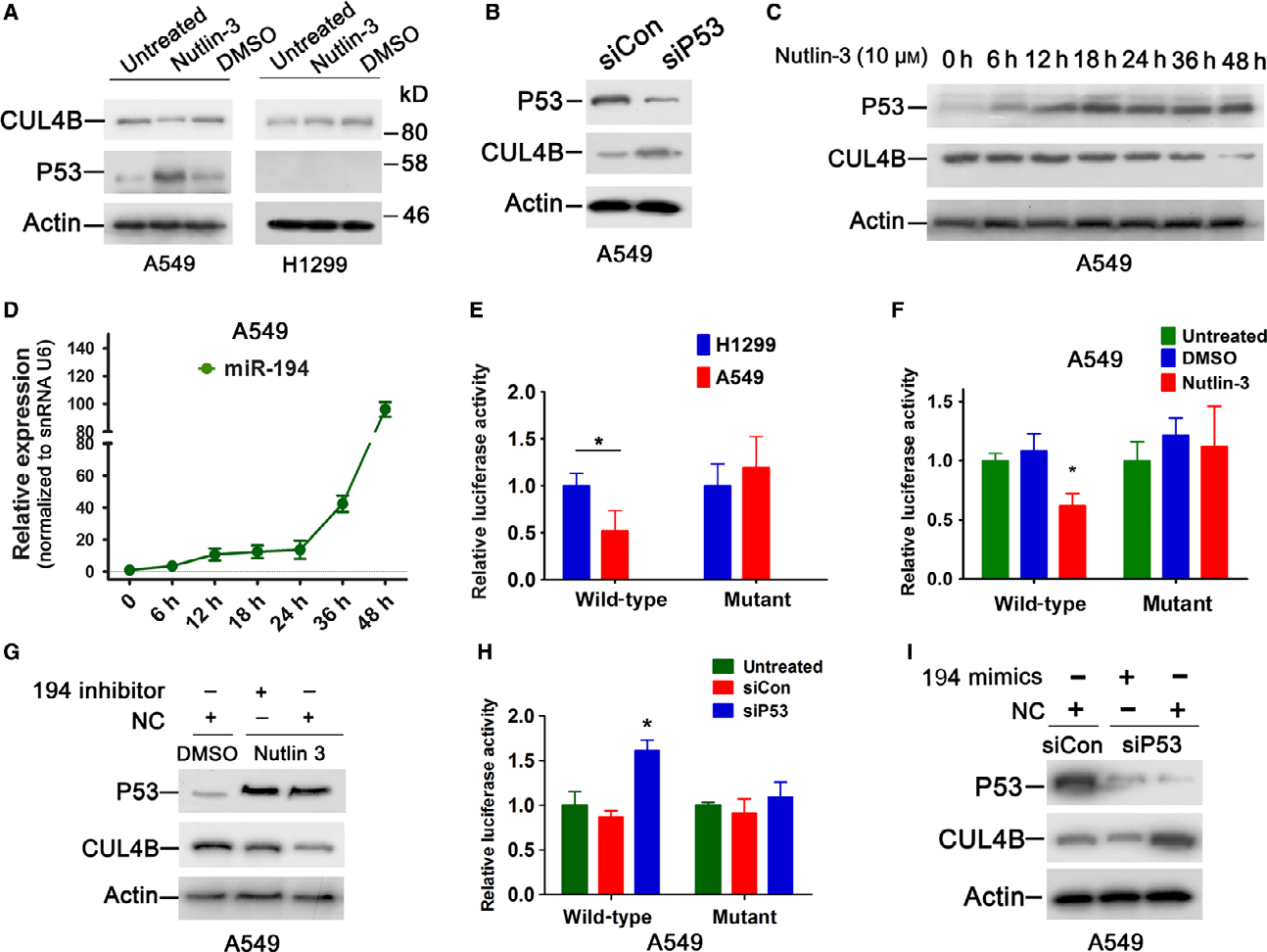
p53 negatively regulates CUL4B expression by transactivating miR‐194. (A) Western blot showing CUL4B and p53 protein levels in A549 and H1299 cells treated with or without 10 μm nutlin‐3 for 60 h. (B) A549 cells were transiently transfected with p53 siRNA or control siRNA. Seventy‐two hours later, CUL4B protein levels were determined. (C, D) A549 cells were treated with 10 μm nutlin‐3 and harvested at the indicated time points. Protein levels were determined by western blot **(**C**)**, and expression of pri‐miR‐194 was determined by real‐time PCR **(**D**)**. Bars represent SEM. (E) Wild‐type or mutant pmir‐GLO‐CUL4B 3′‐UTR plasmids were transiently transfected into A549 and H1299 cells. Forty‐eight hours later, luciferase assays were performed. Bars represent SD. *P < 0.05. (F) A549 cells were transfected with wild‐type or mutant reporter vectors containing CUL4B 3′‐UTR. Twelve hours later, cells were then treated with or without 10 μm nutlin‐3 for another 60 h, and luciferase assays were performed. *P < 0.05. (G) A549 cells treated with nutlin‐3 or DMSO were transfected with miR‐194 inhibitor or control RNA. Sixty hours later, protein levels were determined. (H) p53 or control siRNA was cotransfected with wild‐type or mutant reporter vectors containing CUL4B 3′‐UTR into A549 cells, and luciferase assay was performed. Bars represent SD. *P < 0.05 versus luciferase activity of siCon cells. (I) p53 or control siRNA was cotransfected with miR‐194 mimics or control RNA into A549 cells. Seventy‐two hours later, CUL4B protein levels were determined. Statistical comparisons were made using two‐tailed unpaired *t*‐test (E, F, and H).

Because miR‐194 is transcriptionally upregulated by p53 (Braun *et al*., 2008; Pichiorri *et al*., 2010; Sundaram *et al*., 2011), we next tested whether CUL4B expression was affected by the function of p53. As expected, nutlin‐3 treatment resulted in a reduction in CUL4B protein (Fig. 4A). Moreover, miR‐194 was upregulated by p53 activation (Fig. S3A). In contrast, CUL4B protein level was not altered by nutlin‐3 treatment in p53‐null H1299 cells (Fig. 4A). Furthermore, miR‐194 was downregulated, while the protein levels of CUL4B were increased in p53‐depleted A549 cells (Fig. 4B and Fig. S3B).

We next determined whether the negative regulation of CUL4B by p53 was mediated by miR‐194. A549 cells were treated with nutlin‐3, and the levels of p53, CUL4B, and miR‐194 were examined at different time points thereafter. As shown in Fig. 4C, p53 level was increased 6 h after nutlin‐3 treatment. In contrast, the primary miR‐194 transcripts exhibited a delayed response to p53 activation. They were only mildly increased at 24 h, but were increased approximately 100‐fold at 48 h (Fig. 4D). Interestingly, the spike in miR‐194 expression coincided with the drop in CUL4B protein level (Fig. 4C). The luciferase assay showed that the activity of the reporter containing the CUL4B 3′‐UTR in H1299 cells was much higher than that in A549 cells (Fig. 4E), which expressed higher level of miR‐194 (Fig. S3C). Correspondingly, H1299 cells expressed higher level of CUL4B protein than A549 cells (Fig. S3D). In contrast, no obvious difference in luciferase activity of mutant CUL4B 3′‐UTR was observed between these two cell lines (Fig. 4E). We next analyzed the effect of nutlin‐3 on the activity of 3′‐UTR of CUL4B. As shown in Fig. 4F, the activity of the luciferase reporter containing wild‐type, but not mutant, CUL4B 3′‐UTR was significantly decreased after treatment with nutlin‐3 in A549 cells. In addition, transfection with anti‐miR‐194 inhibitor could effectively block the decrease in CUL4B protein and luciferase activity in nutlin‐3‐treated cells (Figs 4G and S3E). Accordingly, the luciferase activity of wild‐type, but not the mutant, CUL4B 3′‐UTR was significantly increased in p53 knockdown A549 cells (Fig. 4H), and transfection with miR‐194 mimics blocked the increase in CUL4B protein caused by p53 RNAi (Fig. 4I). Together, these data indicate that CUL4B is negatively regulated by the p53–miR‐194 axis.

### 3.5 CUL4B represses transcription of miR‐194 in NSCLC cells

**Figure 5:**
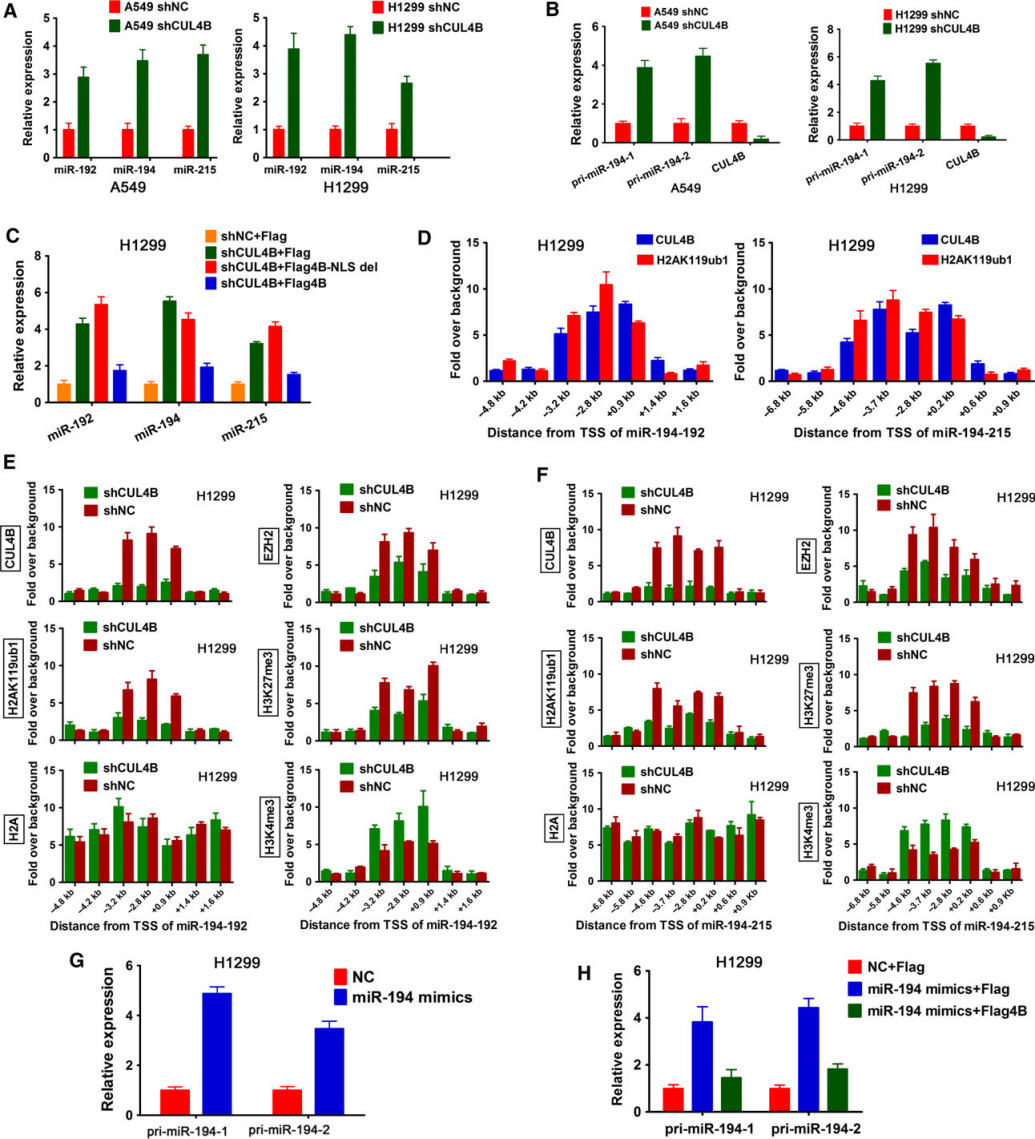
Cullin 4B epigenetically represses transcription of miR‐194. (A) Indicated miRNA levels in shCUL4B and shNC cells were determined using real‐time PCR. Bars represent SEM. (B) Expression of pri‐miR‐194‐1 and pri‐miR‐194‐2 was measured in indicated cells using real‐time PCR. Bars represent SEM. (C) shCUL4B and shNC H1299 cells were transfected with indicated flag‐tagged CUL4B expressing plasmids or empty control plasmid pCMV‐Tag‐2B (Flag). Seventy‐two hours later, levels of miR‐192, miR‐194, and miR‐215 were determined by real‐time PCR. Bars represent SEM. (D) ChIP‐qPCR analysis of CUL4B and H2AK119ub1 at promoters of miR‐194 clusters in H1299 cells. Bars represent SEM. (E, F) ChIP‐qPCR analysis of indicated proteins at promoters of miR‐194 clusters. Bars represent SEM. (G) Real‐time PCR analysis of pri‐miR‐194‐1 and pri‐miR‐194‐2 expression in H1299 cells transfected with miR‐194 mimics or negative control RNA. Bars represent SEM. (H) miR‐194 mimics or NC was cotransfected with control vector pCMV‐Tag‐2B (Flag) or flag‐tagged CUL4B expression vector (Flag4B) into H1299 cells, and the expression of indicated mRNAs was determined using real‐time PCR. Bars represent SEM.

High‐throughput qPCR analyses of cancer‐related miRNAs in CUL4B knockdown A549 and control cells showed that much more miRNAs were upregulated (≥ twofold) than those that were downregulated in CUL4B knockdown cells (Appendix S1), suggesting that CUL4B may primarily function to repress the biogenesis of miRNAs. Interestingly, miR‐194 was also among the significantly upregulated miRNAs in CUL4B knockdown cells. In addition, expression levels of miR‐192 and miR‐215, which reside together with miR‐194 in the miR‐192‐194‐2 cluster and the miR‐215‐194‐1 cluster, respectively, were also elevated in CUL4B RNAi cells. These changes were further confirmed using qPCR (Fig. 5A). The observations that all members of miR‐192‐194‐2 and miR‐215‐194‐1 clusters were upregulated by CUL4B knockdown prompted us to examine the expression of pri‐miRNA of these two clusters. As shown in Fig. 5B, the level of pri‐miR‐194‐1/2 was significantly increased in CUL4B knockdown cells. Furthermore, transfection of wild‐type, but not control or nuclear localization signal (NLS)‐deleted, CUL4B expression vectors attenuated the increased expression of these miRNAs caused by CUL4B knockdown (Fig. 5C). Together, these data suggest that CUL4B may repress the initial step of miR‐194 biogenesis.

Cullin 4B was shown to function as a transcriptional corepressor through catalyzing H2AK119 monoubiquitination (Hu *et al*., 2012). ChIP showed that CUL4B could directly bind to the promoters of the miR‐192‐194‐2 and miR‐215‐194‐1 clusters, and this binding was accompanied by increased H2AK119ub1 (Figs 5D and S4A). Consistent with these results, RNAi of CUL4B significantly reduced the levels of CUL4B binding to the promoters as well as those of H2AK119ub1 (Fig. 5E,F).

We previously reported that CUL4B could act in concert with PRC2 to repress target genes (Hu *et al*., 2012). We therefore tested whether CUL4B also repressed transcription of miR‐194 through EZH2, the catalytic component in PRC2. Indeed, inhibition of EZH2 with DZNep also increased levels of miR‐194 clusters (Fig. S4B). ChIP assay revealed that the promoter regions of miR‐194 clusters are highly enriched for EZH2 and H3K27me3, with occupancy sites overlapping with those of CUL4B (Fig. 5E,F). In addition, knockdown of CUL4B markedly reduced the enrichment of EZH2, as well as the associated H3K27me3 mark in these regions (Fig. 5E,F). Collectively, these results suggest that CUL4B can repress the transcription of miR‐194 clusters by promoting H2AK119 monoubiquitination and consequently the recruitment of EZH2 and H3K27 trimethylation.

The repression of miR‐194 by CUL4B together with the finding that CUL4B is a direct target of miR‐194 suggests that miR‐194 and CUL4B may form a double‐negative feedback loop. As expected, transfection with miR‐194 mimics efficiently elevated pri‐miR‐194 (Fig. 5G). Similar results were obtained for mature miR‐192 and miR‐215 (Fig. S4C). Importantly, this upregulation was dramatically attenuated by cotransfection of CUL4B expression vector (Fig. 5H). Collectively, these data indicate that miR‐194 and CUL4B can regulate each other in a double‐negative feedback loop.

### 3.6 miR‐194 repression mediates the oncogenic activity of CUL4B

**Figure 6:**
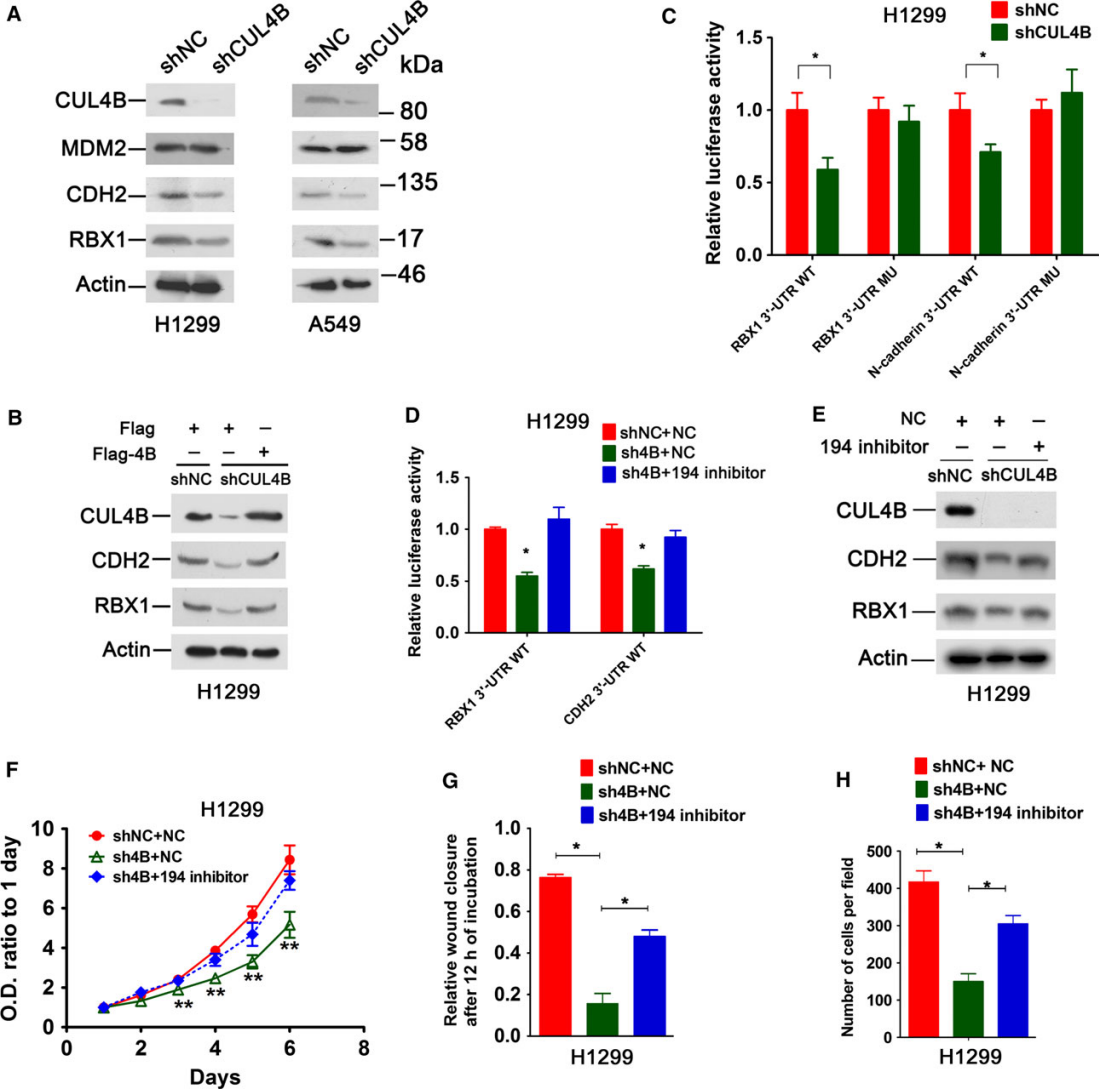
miR‐194–CRL4B negative feedback loop operates NSCLC cells. (A) Western blot analysis of indicated proteins in shNC and shCUL4B H1299 and A549 cells. (B) shCUL4B and shNC H1299 cells were transiently transfected with flag‐tagged CUL4B expression (Flag‐4B) or empty control vector pCMV‐Tag‐2B (Flag). Seventy‐two hours later, cells were harvested and analyzed by western blot. (C) shNC and shCUL4B H1299 cells were transiently transfected with luciferase reporter vectors containing wild‐type or mutant RBX1 or N‐cadherin (CDH2) 3′‐UTR. Forty‐eight hours later, luciferase assays were performed. The luciferase activity of each vector in shNC cells was set as 1. Bars represent SD. *P <* *0.05. (D) Luciferase reporter activities in shNC and shCUL4B H1299 cells transfected with miR‐194 inhibitor or control RNA (NC). Bars represent SD. *P <* *0.05 versus shNC cells transfected with control RNA. (E) shNC and shCUL4B H1299 cells were transiently transfected with miR‐194 inhibitor or control RNA, and protein levels were analyzed. (F–H) Cells were transiently transfected with miR‐194 inhibitor or control RNA. MTT assay (F), wound‐healing assay (G), and Matrigel invasion assay (H) were performed. Bars represent SD. *P <* *0.05. **P <* *0.001. Statistical comparisons were made using two‐tailed unpaired t‐test (C to D, F to H).

Because CUL4B suppresses miR‐194 expression, we predicted that miR‐194 target genes may be downregulated in CUL4B knockdown cells due to the derepression of miR‐194. Indeed, knockdown of CUL4B significantly reduced the levels of RBX1 and N‐cadherin (CDH2), two targets of miR‐194 (Fig. 6A). Moreover, ectopic expression of CUL4B could effectively restore the RBX1 and N‐cadherin levels in CUL4B knockdown cells, suggesting that CUL4B positively regulates expression of RBX1 and N‐cadherin (Fig. 6B).

The luciferase assay showed that knockdown of CUL4B markedly suppressed the activities of reporters containing RBX1 and N‐cadherin wild‐type 3′‐UTR, whereas reporters carrying the mutant 3′‐UTR were unresponsive to CUL4B knockdown (Fig. 6C). Additionally, inhibition of miR‐194 could reverse the decreased activity of RBX1 or N‐cadherin wild‐type 3′‐UTR‐containing reporter (Fig. 6D). In line with these results, inhibition of miR‐194 was able to rescue the decreased levels of RBX1 and N‐cadherin protein in shCUL4B cells (Fig. 6E). Next, we explored the role of miR‐194 repression by CUL4B in maintaining the malignancy of NSCLC cells. As shown in Fig. 6F–H, inhibition of miR‐194 remarkably restored the proliferation, mobility, and invasiveness in CUL4B knockdown H1299 cells, indicating that CUL4B contributes to oncogenesis of NSCLC at least partly through repression of miR‐194.

## 4 Discussion

Cullin 4B‐RING‐based E3 ubiquitin ligases has been shown to epigenetically repress gene expression through catalyzing H2AK119 monoubiquitination (Hu *et al*., 2012). It possesses oncogenic properties by repressing tumor suppressors in a variety of human cancers (Hu *et al*., 2012; Ji *et al*., 2014; Yang *et al*., 2015; Yuan *et al*., 2015). This study shows that CUL4B is upregulated in human lung cancer tissues and contributes to proliferation, migration, and invasion of NSCLC cells. Importantly, we demonstrated an important role of miR‐194 in the posttranscriptional regulation of CUL4B in lung cancer. miR‐194 can directly target CUL4B, inhibit its translation, and attenuate the phenotypes caused by CUL4B overexpression. Moreover, miR‐194 expression is negatively correlated with CUL4B protein levels in lung cancer tissues, and miR‐194 is epigenetically repressed by the CRL4B complex assembled by CUL4B. To our knowledge, this is the first study to demonstrate a mechanism by which CUL4B is upregulated posttranscriptionally in cancer tissues.

Many studies showed that miR‐194 is downregulated and functions as a tumor suppressor by downregulating its target genes such as N‐cadherin (Meng *et al*., 2010), AKT2 (Zhao *et al*., 2014) and BMI1 (Dong *et al*., 2011) in a variety of cancers (Khella *et al*., 2013; Meng *et al*., 2010; Senanayake *et al*., 2012; Wang *et al*., 2015; Zhao *et al*., 2014; Zhu *et al*., 2016). Through directly inhibiting its targets, miR‐194 suppresses proliferation and metastasis of cancer cells (Dong *et al*., 2011; Han *et al*., 2014; Meng *et al*., 2010; Wang *et al*., 2015; Wu *et al*., 2014; Zhu *et al*., 2016). Mature miR‐194 is derived from miR‐194‐2/192 cluster on chromosome 1q41 and miR‐194‐1/215 cluster on chromosome 11q13.1 (Pichiorri *et al*., 2010). Like miR‐194, miR‐192 and miR‐215 are also found to be tumor suppressor genes in many cancers including NSCLC (Hou *et al*., 2015; Jin *et al*., 2015; Khella *et al*., 2013; Lian *et al*., 2016). Because clustered miRNAs are usually cotranscribed, miR‐194, miR‐192, and miR‐215 may thus be subjected to the same regulation at transcription level. While the promoters of these two clusters were shown to be p53‐responsive in several cancer lines (Braun *et al*., 2008; Pichiorri *et al*., 2010), another study reported that inhibition of p53 did not prevent the induction of miR‐194‐2/192 by aristolochic acid in proximal tubular epithelial cells (Jenkins *et al*., 2014), indicating that the mechanisms by which expression of miR‐194 clusters is regulated are diverse and may operate in a context‐specific way. Our findings that CUL4B could bind to and repress the two miR‐194 clusters in both p53 wild‐type (A549) and p53‐null (H1299) NSCLC cells suggest that CUL4B may repress miR‐194 transcription independent of p53. However, by upregulating miR‐194, p53 can presumably offset the repressive effect of CUL4B.

Cullin 4B‐RING‐based E3 ubiquitin ligase was found to function as a transcriptional repressor by catalyzing histone H2A monoubiquitination at lysine 119, which facilitates the recruitment of PRC2 to chromatin and in turn catalyzes trimethylation of histone H3 lysine 27 (Hu *et al*., 2012). Accordingly, we demonstrated that CUL4B was critical for H2AK119ub1, EZH2 recruitment to and the consequent H3K27me3 at promoters of the two miR‐194 clusters. It should be mentioned that many miRNAs, such as miR‐30d, miR‐31, miR‐200, miR‐101, and let‐7c, were found to be repressed by EZH2 in various types of tumors (Au *et al*., 2012; Cao *et al*., 2011; Wang *et al*., 2014; Yamagishi *et al*., 2012; Zhang *et al*., 2014a,b). Two recent studies also implicated EZH2 in the regulation of miR‐194 expression (Hibino *et al*., 2014; Zhao *et al*., 2014). Our results described here substantiated the paradigm that miRNAs can be epigenetically repressed, via CRL4B and PRC2 complexes.

**Figure 7:**
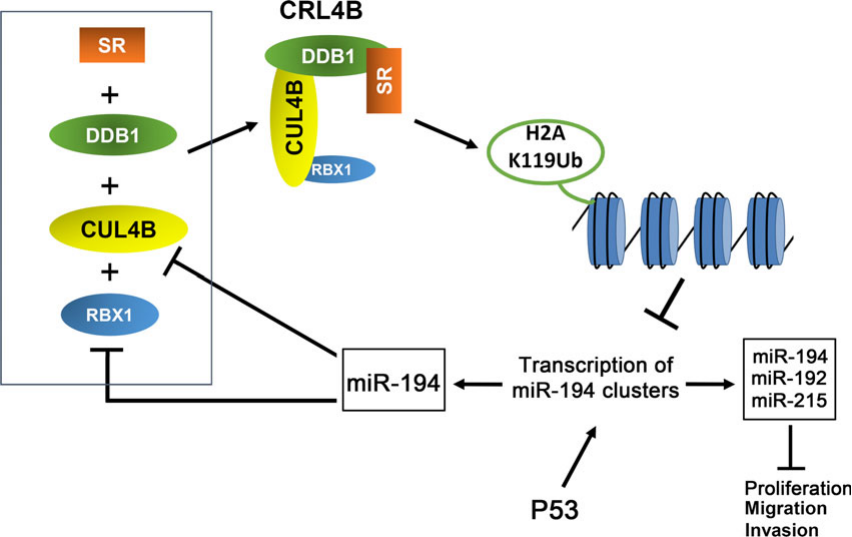
Model for a double‐negative feedback loop between CRL4B and miR‐194 in NSCLC cells. p53 transactivates the transcription of miR‐194 clusters, leading to increased production of miR‐192, miR‐194, and miR‐215. These miRNAs negatively regulate a large number of oncogenic target genes and inhibit proliferation, migration, and invasion of NSCLC cells. The E3 ubiquitin ligase CRL4B epigenetically represses miR‐194 clusters by catalyzing monoubiquitination at H2AK119 at the promoters. Meanwhile, miR‐194 could repress CRL4B by directly binding to the 3′‐UTR of CUL4B and RBX1 mRNA, encoding two subunits of CRL4B. Thus, this system forms a double‐negative feedback loop in lung cancer tumorigenesis, which would enable a feed‐forward upregulation of either of the two antagonizing members when its repression is alleviated. SR, substrate receptor.

The reciprocal negative feedback loops formed by miRNA and their targets may function to fine‐tune gene expression (Fang and Gao, 2014). For example, PRC2 epigenetically represses miR‐101 in a c‐Myc‐mediated manner. miR‐101, in turn, inhibits the expression of two subunits of PRC2, thus creating a double‐negative feedback loop in hepatocarcinogenesis (Wang *et al*., 2014). Our current findings indicate that CUL4B and miR‐194 reciprocally repress each other directly. These findings, together with the result that RBX1, a subunit of CRL4B that is overexpressed in a number of human tumors (Jia *et al*., 2009), is also targeted by miR‐194 (Chen *et al*., 2015), established that CRL4B and miR‐194 form a negative feedback loop in NSCLC cells (Fig. 7). This loop would enable a feed‐forward upregulation of either of the two antagonizing members when its repression is alleviated. The delicate balance, which may exist in normal tissues, appears to tip toward CRL4B in NSCLC, leading to further CUL4B upregulation and miR‐194 repression.

In summary, this study shows that CUL4B is upregulated in and required for the oncogenicity of NSCLC cells. Moreover, we also showed that CUL4B was a direct target of miR‐194 and CUL4B could epigenetically repress expression of miR‐194 by monoubiquitination of H2AK119 and trimethylation of lysine 27 on histone 3 on the miR‐194 promoter, thus creating a double‐negative feedback loop between miR‐194 and CRL4B in lung cancer tumorigenesis. Taken together, our data highlight the important role of CUL4B in lung cancer progression and establish a complex, microRNA‐mediated regulation of CUL4B protein levels. Given the important roles of miR‐194 cluster genes in repressing CUL4B and inhibiting tumor progression in NSCLC, miR‐194 mimics should be explored as a potential therapeutic agent.

## Author contributions

The authors contributed to this study in the following way: Conception and design: JM, YZ, CS and YG; performed experiments: JM, YZ, XL, JL, XL, HZ and XY; analysis and interpretation of data: JM, YZ, CS and YG; writing, review and/or revision of the manuscript: YZ, CS and YG; Administrative, technical, or material support: HH, BJ, BH; Study supervision: YG.

## Supporting information


**Appendix S1.** Supplementary results.

**Fig. S1.** Knockdown of CUL4B inhibits proliferation, migration and invasion of NSCLC cells.
**Fig. S2.** CUL4B is a target of miR‐194.
**Fig. S3.** p53 downregulates CUL4B by transactivating miR‐194.
**Fig. S4.** CUL4B represses miR‐194 expression.

**Table S1.** Primer sequences used for qPCR.
**Table S2.** Primary antibodies for western blot.
**Table S3.** Primer sequences used for site‐directed mutagenesis.
**Table S4.** Primer sequences used for ChIP‐qPCR.
**Table S5.** Primary antibodies for ChIP.
**Table S6.** Correlation of the High CUL4B Expression with Clinicopathological Characteristics.
