# Supplementary material for: Dysregulation of the miR‐194–CUL4B negative feedback loop drives tumorigenesis in non‐small‐cell lung carcinoma
Source: Mol Oncol. 2017 Feb 21;11(3):305–19. doi: 10.1002/1878-0261.12038 (PMC5527444; doi:10.1002/1878-0261.12038)
Supplement: Supplementary file 1 — Appendix S1. Supplementary results. [file MOL2-11-305-s001.pdf]

Supplementary results

Plate	miRNA Name	AVG ΔCt (Ct(GOI) - Ave Ct (HKG))		2^-ΔCt		Fold Difference	Fold Up- or Down-Regulation
		shCUL4B	shNC	shCUL4B	shNC	shCUL4B /shNC	shCUL4B /shNC
Plate 1	hsa-miR-1	6.78	8.33	9.1E-03	3.1E-03	2.93	2.93
	hsa-miR-101-3p	5.37	5.97	2.4E-02	1.6E-02	1.52	1.52
	hsa-miR-105-5p	6.78	8.33	9.1E-03	3.1E-03	2.93	2.93
	hsa-miR-106b-5p	6.78	7.15	9.1E-03	7.1E-03	1.29	1.29
	hsa-miR-122-5p	6.78	8.33	9.1E-03	3.1E-03	2.93	2.93
	hsa-miR-16-5p	1.98	1.77	2.5E-01	2.9E-01	0.86	-1.16
	hsa-miR-124-3p	6.55	7.63	1.1E-02	5.1E-03	2.11	2.11
	hsa-miR-125a-5p	0.74	0.86	6.0E-01	5.5E-01	1.09	1.09
	hsa-miR-125b-5p	-1.71	-1.81	3.3E+00	3.5E+00	0.93	-1.07
	hsa-miR-137	6.78	8.33	9.1E-03	3.1E-03	2.93	2.93
	hsa-miR-139-5p	5.27	6.16	2.6E-02	1.4E-02	1.85	1.85
	hsa-miR-145-5p	6.78	8.33	9.1E-03	3.1E-03	2.93	2.93
	hsa-miR-149-5p	5.34	5.59	2.5E-02	2.1E-02	1.19	1.19
	hsa-miR-153	6.78	8.33	9.1E-03	3.1E-03	2.93	2.93
	hsa-miR-154-5p	6.78	7.07	9.1E-03	7.5E-03	1.22	1.22
	hsa-miR-182-5p	6.78	7.78	9.1E-03	4.6E-03	2.00	2.00
	hsa-miR-183-5p	6.78	8.33	9.1E-03	3.1E-03	2.93	2.93
	hsa-miR-190a	6.78	8.33	9.1E-03	3.1E-03	2.93	2.93
	hsa-miR-219-5p	6.78	8.33	9.1E-03	3.1E-03	2.93	2.93
	hsa-miR-22-3p	6.78	7.96	9.1E-03	4.0E-03	2.27	2.27
	hsa-miR-26a-5p	4.58	3.51	4.2E-02	8.8E-02	0.48	-2.10
	hsa-miR-29b-3p	6.78	8.33	9.1E-03	3.1E-03	2.93	2.93
	hsa-miR-30c-5p	4.20	4.95	5.5E-02	3.2E-02	1.68	1.68
	hsa-miR-9-5p	6.78	8.33	9.1E-03	3.1E-03	2.93	2.93
	hsa-miR-99b-5p	3.31	3.09	1.0E-01	1.2E-01	0.86	-1.16
	hsa-miR-129-5p	2.39	3.85	1.9E-01	7.0E-02	2.75	2.75
	hsa-miR-15a-5p	6.78	6.86	9.1E-03	8.6E-03	1.06	1.06
	hsa-miR-15b-5p	1.55	1.52	3.4E-01	3.5E-01	0.98	-1.02
	hsa-miR-21-5p	0.54	0.22	6.9E-01	8.6E-01	0.81	-1.24
	hsa-miR-210	4.46	4.98	4.6E-02	3.2E-02	1.43	1.43
	hsa-miR-212-3p	4.67	5.22	3.9E-02	2.7E-02	1.46	1.46
	hsa-miR-24-3p	3.36	3.02	9.8E-02	1.2E-01	0.79	-1.27
	hsa-miR-30b-5p	4.00	3.77	6.3E-02	7.4E-02	0.85	-1.17

	hsa-miR-34b-3p	6.78	8.33	9.1E-03	3.1E-03	**2.93**	**2.93**
	hsa-miR-372	6.78	8.33	9.1E-03	3.1E-03	**2.93**	**2.93**
	hsa-miR-423-3p	4.77	4.20	3.7E-02	5.5E-02	0.67	-1.48
	hsa-miR-126-3p	6.78	8.33	9.1E-03	3.1E-03	**2.93**	**2.93**
	hsa-miR-132-3p	6.78	8.33	9.1E-03	3.1E-03	**2.93**	**2.93**
	hsa-miR-134	6.28	6.41	1.3E-02	1.2E-02	1.09	1.09
	hsa-miR-136-5p	6.78	8.33	9.1E-03	3.1E-03	**2.93**	**2.93**
	hsa-miR-138-5p	4.60	3.60	4.1E-02	8.3E-02	0.50	-2.00
	hsa-miR-140-5p	6.78	8.33	9.1E-03	3.1E-03	**2.93**	**2.93**
	hsa-miR-142-3p	6.78	8.33	9.1E-03	3.1E-03	**2.93**	**2.93**
	hsa-miR-142-5p	6.78	8.33	9.1E-03	3.1E-03	**2.93**	**2.93**
	hsa-miR-143-3p	6.78	8.33	9.1E-03	3.1E-03	**2.93**	**2.93**
	hsa-miR-152	6.78	8.33	9.1E-03	3.1E-03	**2.93**	**2.93**
	hsa-miR-185-5p	5.44	5.86	2.3E-02	1.7E-02	1.34	1.34
	hsa-miR-186-5p	6.78	8.33	9.1E-03	3.1E-03	**2.93**	**2.93**
	hsa-miR-187-3p	5.47	7.15	2.3E-02	7.1E-03	**3.20**	**3.20**
	hsa-miR-188-5p	4.84	6.57	3.5E-02	1.1E-02	**3.32**	**3.32**
	hsa-miR-191-5p	2.62	1.91	1.6E-01	2.7E-01	0.61	-1.64
	hsa-miR-193a-3p	6.78	8.33	9.1E-03	3.1E-03	**2.93**	**2.93**
	hsa-miR-194-5p	6.78	8.33	9.1E-03	3.1E-03	**2.93**	**2.93**
	hsa-miR-195-5p	6.77	6.33	9.2E-03	1.2E-02	0.74	-1.36
	hsa-miR-205-5p	6.78	8.33	9.1E-03	3.1E-03	**2.93**	**2.93**
	hsa-miR-206	6.67	8.07	9.9E-03	3.7E-03	**2.64**	**2.64**
	hsa-miR-216a	6.78	8.33	9.1E-03	3.1E-03	**2.93**	**2.93**
	hsa-miR-218-5p	6.78	8.33	9.1E-03	3.1E-03	**2.93**	**2.93**
	hsa-miR-221-3p	4.85	3.74	3.5E-02	7.5E-02	0.46	**-2.16**
	hsa-miR-223-3p	6.78	8.33	9.1E-03	3.1E-03	**2.93**	**2.93**
	hsa-miR-25-3p	0.65	0.65	6.4E-01	6.4E-01	1.00	-1.00
	hsa-miR-296-5p	6.78	8.33	9.1E-03	3.1E-03	**2.93**	**2.93**
	hsa-miR-302a-3p	6.78	8.33	9.1E-03	3.1E-03	**2.93**	**2.93**
	hsa-miR-32-5p	6.78	8.33	9.1E-03	3.1E-03	**2.93**	**2.93**
	hsa-miR-335-5p	6.78	8.33	9.1E-03	3.1E-03	**2.93**	**2.93**
	hsa-miR-34a-5p	6.78	7.84	9.1E-03	4.4E-03	**2.08**	**2.08**
	hsa-miR-373-3p	6.78	8.33	9.1E-03	3.1E-03	**2.93**	**2.93**
	hsa-miR-92a-3p	-1.39	-1.22	2.6E+00	2.3E+00	1.13	1.13
	hsa-miR-93-5p	3.93	3.39	6.6E-02	9.6E-02	0.69	-1.45
	hsa-miR-96-5p	6.78	8.33	9.1E-03	3.1E-03	**2.93**	**2.93**
	hsa-let-7a-5p	-1.91	-3.48	3.7E+00	1.1E+01	0.34	**-2.97**
	hsa-let-7e-5p	-3.67	-3.19	1.3E+01	9.1E+00	1.39	1.39
	hsa-let-7f-5p	0.65	0.38	6.4E-01	7.7E-01	0.84	-1.20
	hsa-miR-217	6.78	7.64	9.1E-03	5.0E-03	1.82	1.82

	hsa-miR-452-5p	6.78	8.33	9.1E-03	3.1E-03	**2.93**	**2.93**
	hsa-miR-100-5p	6.78	-0.47	9.1E-03	1.4E+00	**0.01**	**-151.17**
	hsa-miR-451a	6.78	8.33	9.1E-03	3.1E-03	**2.93**	**2.93**
	hsa-miR-508-5p	6.78	8.33	9.1E-03	3.1E-03	**2.93**	**2.93**
	hsa-miR-572	4.50	4.54	4.4E-02	4.3E-02	1.03	1.03
	hsa-miR-99a-5p	2.42	2.59	1.9E-01	1.7E-01	1.13	1.13
	hsa-miR-491-5p	6.78	8.33	9.1E-03	3.1E-03	**2.93**	**2.93**
	hsa-miR-130a-3p	6.78	7.33	9.1E-03	6.2E-03	1.46	1.46
	hsa-miR-30a-5p	6.78	8.33	9.1E-03	3.1E-03	**2.93**	**2.93**
	hsa-miR-144-3p	6.78	8.33	9.1E-03	3.1E-03	**2.93**	**2.93**
	hsa-miR-34c-5p	6.75	8.36	9.3E-03	3.1E-03	**3.05**	**3.05**
	hsa-miR-30e-5p	6.75	8.36	9.3E-03	3.1E-03	**3.05**	**3.05**
	hsa-miR-199b-5p	6.75	8.36	9.3E-03	3.1E-03	**3.05**	**3.05**
	hsa-miR-193b-3p	3.30	2.24	1.0E-01	2.1E-01	0.48	**-2.09**
	hsa-miR-31-5p	5.67	6.02	2.0E-02	1.5E-02	1.27	1.27
	hsa-miR-30d-5p	6.75	8.11	9.3E-03	3.6E-03	**2.56**	**2.56**
	hsa-miR-302f	6.75	8.36	9.3E-03	3.1E-03	**3.05**	**3.05**
	hsa-miR-497-5p	3.50	2.92	8.8E-02	1.3E-01	0.67	-1.50
**Plate 2**	hsa-miR-514a-3p	6.75	8.36	9.3E-03	3.1E-03	**3.05**	**3.05**
	hsa-miR-26b-5p	5.66	5.84	2.0E-02	1.7E-02	1.13	1.13
	hsa-miR-302e	6.75	8.36	9.3E-03	3.1E-03	**3.05**	**3.05**
	hsa-miR-410	6.75	8.36	9.3E-03	3.1E-03	**3.05**	**3.05**
	hsa-miR-429	6.75	8.36	9.3E-03	3.1E-03	**3.05**	**3.05**
	hsa-miR-190b	6.75	8.36	9.3E-03	3.1E-03	**3.05**	**3.05**
	hsa-miR-411-5p	6.75	8.36	9.3E-03	3.1E-03	**3.05**	**3.05**
	hsa-miR-628-5p	6.75	8.05	9.3E-03	3.8E-03	**2.46**	**2.46**
	hsa-miR-561-3p	6.75	8.36	9.3E-03	3.1E-03	**3.05**	**3.05**
	hsa-miR-340-5p	6.75	8.36	9.3E-03	3.1E-03	**3.05**	**3.05**
	hsa-miR-627	4.62	5.79	4.1E-02	1.8E-02	**2.24**	**2.24**
	hsa-miR-7-5p	6.75	7.99	9.3E-03	3.9E-03	**2.36**	**2.36**
	hsa-miR-379-5p	6.75	8.36	9.3E-03	3.1E-03	**3.05**	**3.05**
	hsa-miR-520a-5p	5.57	5.00	2.1E-02	3.1E-02	0.67	-1.49
	hsa-miR-224-5p	6.27	6.29	1.3E-02	1.3E-02	1.01	1.01
	hsa-miR-325	6.75	8.36	9.3E-03	3.1E-03	**3.05**	**3.05**
	hsa-miR-563	6.75	8.08	9.3E-03	3.7E-03	**2.51**	**2.51**
	hsa-miR-220a	6.75	8.36	9.3E-03	3.1E-03	**3.05**	**3.05**
	hsa-miR-513a-5p	6.75	8.16	9.3E-03	3.5E-03	**2.65**	**2.65**

	hsa-miR-203	6.75	8.36	9.3E-03	3.1E-03	**3.05**	**3.05**
	hsa-miR-28-5p	6.07	7.03	1.5E-02	7.7E-03	1.94	1.94
	hsa-miR-329	6.75	8.36	9.3E-03	3.1E-03	**3.05**	**3.05**
	hsa-miR-365a-3p	4.75	3.90	3.7E-02	6.7E-02	0.55	-1.81
	hsa-miR-222-3p	6.75	7.90	9.3E-03	4.2E-03	**2.21**	**2.21**
	hsa-miR-491-3p	6.75	8.36	9.3E-03	3.1E-03	**3.05**	**3.05**
	hsa-miR-146b-5p	6.75	8.36	9.3E-03	3.1E-03	**3.05**	**3.05**
	hsa-miR-151a-5p	2.35	2.02	2.0E-01	2.5E-01	0.79	-1.26
	hsa-miR-582-5p	6.75	8.36	9.3E-03	3.1E-03	**3.05**	**3.05**
	hsa-miR-337-5p	6.75	7.20	9.3E-03	6.8E-03	1.36	1.36
	hsa-miR-628-3p	4.39	4.33	4.8E-02	5.0E-02	0.96	-1.04
	hsa-miR-381	6.75	8.36	9.3E-03	3.1E-03	**3.05**	**3.05**
	hsa-miR-516a-3p	6.75	8.36	9.3E-03	3.1E-03	**3.05**	**3.05**
	hsa-miR-337-3p	6.75	8.36	9.3E-03	3.1E-03	**3.05**	**3.05**
	hsa-miR-377-3p	6.75	8.36	9.3E-03	3.1E-03	**3.05**	**3.05**
	hsa-miR-495	6.75	8.36	9.3E-03	3.1E-03	**3.05**	**3.05**
	hsa-miR-128	6.75	7.95	9.3E-03	4.1E-03	**2.29**	**2.29**
	hsa-miR-513a-3p	6.75	8.36	9.3E-03	3.1E-03	**3.05**	**3.05**
	hsa-miR-582-3p	6.75	8.36	9.3E-03	3.1E-03	**3.05**	**3.05**
	hsa-miR-151a-3p	6.38	5.64	1.2E-02	2.0E-02	0.60	-1.67
	hsa-miR-297	6.75	8.13	9.3E-03	3.6E-03	**2.60**	**2.60**
	hsa-miR-323a-3p	6.75	7.39	9.3E-03	6.0E-03	1.55	1.55
	hsa-miR-378a-3p	6.75	7.80	9.3E-03	4.5E-03	**2.07**	**2.07**
	hsa-miR-299-5p	6.75	7.25	9.3E-03	6.6E-03	1.41	1.41
	hsa-miR-511	6.75	7.63	9.3E-03	5.1E-03	1.84	1.84
	hsa-miR-521	6.75	8.36	9.3E-03	3.1E-03	**3.05**	**3.05**
	hsa-miR-361-5p	6.22	6.83	1.3E-02	8.8E-03	1.52	1.52
	hsa-miR-383	6.75	8.36	9.3E-03	3.1E-03	**3.05**	**3.05**
	hsa-miR-155-5p	3.64	3.50	8.0E-02	8.9E-02	0.91	-1.10
	hsa-miR-508-3p	6.75	8.36	9.3E-03	3.1E-03	**3.05**	**3.05**
	hsa-miR-525-5p	6.75	8.36	9.3E-03	3.1E-03	**3.05**	**3.05**
	hsa-miR-648	6.75	8.36	9.3E-03	3.1E-03	**3.05**	**3.05**
	hsa-miR-28-3p	5.39	5.31	2.4E-02	2.5E-02	0.94	-1.06
	hsa-miR-342-5p	6.75	8.36	9.3E-03	3.1E-03	**3.05**	**3.05**

	hsa-miR-219-2-3p	6.75	8.36	9.3E-03	3.1E-03	**3.05**	**3.05**
	hsa-miR-220c	6.75	8.36	9.3E-03	3.1E-03	**3.05**	**3.05**
	hsa-miR-147a	2.23	3.53	2.1E-01	8.7E-02	**2.46**	**2.46**
	hsa-miR-371a-5p	3.87	5.88	6.8E-02	1.7E-02	**4.02**	**4.02**
	hsa-miR-367-3p	6.75	8.36	9.3E-03	3.1E-03	**3.05**	**3.05**
	hsa-miR-95	6.75	8.36	9.3E-03	3.1E-03	**3.05**	**3.05**
	hsa-miR-510	6.75	7.98	9.3E-03	4.0E-03	**2.34**	**2.34**
	hsa-miR-33a-5p	6.75	8.36	9.3E-03	3.1E-03	**3.05**	**3.05**
	hsa-miR-455-5p	6.75	8.36	9.3E-03	3.1E-03	**3.05**	**3.05**
	hsa-miR-608	6.75	8.36	9.3E-03	3.1E-03	**3.05**	**3.05**
	hsa-miR-483-3p	6.75	8.36	9.3E-03	3.1E-03	**3.05**	**3.05**
	hsa-miR-220b	6.75	8.36	9.3E-03	3.1E-03	**3.05**	**3.05**
	hsa-miR-642a-5p	6.75	8.36	9.3E-03	3.1E-03	**3.05**	**3.05**
	hsa-miR-127-5p	6.75	8.36	9.3E-03	3.1E-03	**3.05**	**3.05**
	hsa-miR-338-5p	6.68	7.84	9.8E-03	4.4E-03	**2.23**	**2.23**
	hsa-miR-455-3p	5.84	6.04	1.7E-02	1.5E-02	1.15	1.15
	hsa-miR-613	6.75	8.36	9.3E-03	3.1E-03	**3.05**	**3.05**
	hsa-miR-424-5p	4.52	5.60	4.4E-02	2.1E-02	**2.11**	**2.11**
	hsa-miR-595	6.75	7.93	9.3E-03	4.1E-03	**2.26**	**2.26**
	hsa-miR-489	5.34	5.31	2.5E-02	2.5E-02	0.98	-1.02
	hsa-miR-708-5p	6.75	7.85	9.3E-03	4.3E-03	**2.14**	**2.14**
	hsa-miR-202-3p	3.00	4.00	1.3E-01	6.3E-02	2.00	2.00
	hsa-miR-299-3p	6.75	8.36	9.3E-03	3.1E-03	**3.05**	**3.05**
	hsa-miR-216b	5.55	6.69	2.1E-02	9.7E-03	**2.20**	**2.20**
	hsa-miR-507	6.72	8.46	9.5E-03	2.8E-03	**3.33**	**3.33**
	hsa-miR-532-5p	6.72	7.90	9.5E-03	4.2E-03	**2.26**	**2.26**
	hsa-miR-422a	5.23	5.20	2.7E-02	2.7E-02	0.98	-1.02
	hsa-miR-494	6.20	5.96	1.4E-02	1.6E-02	0.84	-1.18
	hsa-miR-184	6.72	8.46	9.5E-03	2.8E-03	**3.33**	**3.33**
	hsa-miR-493-3p	4.83	6.18	3.5E-02	1.4E-02	**2.54**	**2.54**
	hsa-miR-622	5.52	6.48	2.2E-02	1.1E-02	1.94	1.94
	hsa-miR-500a-5p	3.27	4.08	1.0E-01	5.9E-02	1.75	1.75
	hsa-miR-516a-5p	6.72	8.46	9.5E-03	2.8E-03	**3.33**	**3.33**
	hsa-miR-150-5p	6.72	8.38	9.5E-03	3.0E-03	**3.15**	**3.15**
	hsa-miR-181d	3.73	5.35	7.5E-02	2.5E-02	**3.07**	**3.07**
	hsa-miR-23b-3p	2.99	3.37	1.3E-01	9.7E-02	1.30	1.30
	hsa-miR-129-2-3p	4.38	5.33	4.8E-02	2.5E-02	1.93	1.93

	hsa-miR-140-3p	5.88	6.33	1.7E-02	1.2E-02	1.36	1.36
	hsa-miR-324-5p	6.00	7.09	1.6E-02	7.3E-03	**2.12**	**2.12**
**Plate 3**	hsa-miR-584-5p	5.37	6.66	2.4E-02	9.9E-03	**2.44**	**2.44**
	hsa-miR-338-3p	6.72	8.46	9.5E-03	2.8E-03	**3.33**	**3.33**
	hsa-miR-382-5p	6.72	6.91	9.5E-03	8.3E-03	1.14	1.14
	hsa-miR-501-5p	3.56	4.61	8.5E-02	4.1E-02	**2.07**	**2.07**
	hsa-miR-331-5p	6.72	8.20	9.5E-03	3.4E-03	**2.78**	**2.78**
	hsa-miR-412	6.72	8.46	9.5E-03	2.8E-03	**3.33**	**3.33**
	hsa-miR-605	6.72	8.46	9.5E-03	2.8E-03	**3.33**	**3.33**
	hsa-miR-515-3p	6.41	6.71	1.2E-02	9.6E-03	1.23	1.23
	hsa-miR-518b	6.72	8.46	9.5E-03	2.8E-03	**3.33**	**3.33**
	hsa-miR-214-3p	6.72	8.46	9.5E-03	2.8E-03	**3.33**	**3.33**
	hsa-miR-345-5p	5.90	6.79	1.7E-02	9.0E-03	1.85	1.85
	hsa-miR-361-3p	6.72	8.46	9.5E-03	2.8E-03	**3.33**	**3.33**
	hsa-miR-483-5p	5.72	7.68	1.9E-02	4.9E-03	**3.88**	**3.88**
	hsa-miR-657	6.72	8.46	9.5E-03	2.8E-03	**3.33**	**3.33**
	hsa-miR-371a-3p	6.72	8.46	9.5E-03	2.8E-03	**3.33**	**3.33**
	hsa-miR-34c-3p	6.46	7.27	1.1E-02	6.5E-03	1.75	1.75
	hsa-miR-425-5p	4.59	5.23	4.1E-02	2.7E-02	1.55	1.55
	hsa-miR-486-3p	6.72	6.63	9.5E-03	1.0E-02	0.94	-1.07
	hsa-miR-492	6.72	8.46	9.5E-03	2.8E-03	**3.33**	**3.33**
	hsa-miR-331-3p	6.72	5.22	9.5E-03	2.7E-02	0.35	**-2.83**
	hsa-miR-330-5p	6.72	8.46	9.5E-03	2.8E-03	**3.33**	**3.33**
	hsa-miR-432-5p	4.38	5.27	4.8E-02	2.6E-02	1.85	1.85
	hsa-miR-551a	4.49	4.78	4.4E-02	3.6E-02	1.22	1.22
	hsa-miR-219-1-3p	6.72	7.90	9.5E-03	4.2E-03	**2.26**	**2.26**
	hsa-miR-652-3p	4.70	4.25	3.8E-02	5.3E-02	0.73	-1.37
	hsa-miR-146b-3p	6.72	8.46	9.5E-03	2.8E-03	**3.33**	**3.33**
	hsa-miR-342-3p	5.16	5.16	2.8E-02	2.8E-02	1.00	-1.00
	hsa-miR-423-5p	4.23	3.65	5.3E-02	8.0E-02	0.67	-1.50
	hsa-miR-125a-3p	3.34	4.04	9.9E-02	6.1E-02	1.62	1.62
	hsa-miR-635	4.45	6.65	4.6E-02	1.0E-02	**4.58**	**4.58**
	hsa-miR-885-3p	6.72	8.46	9.5E-03	2.8E-03	**3.33**	**3.33**
	hsa-miR-485-5p	5.14	7.46	2.8E-02	5.7E-03	**4.98**	**4.98**
	hsa-miR-326	5.73	7.31	1.9E-02	6.3E-03	**2.98**	**2.98**
	hsa-miR-188-3p	4.83	4.88	3.5E-02	3.4E-02	1.03	1.03
	hsa-miR-512-5p	6.72	8.46	9.5E-03	2.8E-03	**3.33**	**3.33**
	hsa-miR-127-3p	6.62	6.42	1.0E-02	1.2E-02	0.87	-1.15
	hsa-miR-486-5p	6.72	7.36	9.5E-03	6.1E-03	1.55	1.55

	hsa-miR-503	5.66	6.54	2.0E-02	1.1E-02	1.84	1.84
	hsa-miR-525-3p	6.22	7.69	1.3E-02	4.8E-03	**2.76**	**2.76**
	hsa-miR-542-5p	3.40	5.66	9.5E-02	2.0E-02	**4.78**	**4.78**
	hsa-miR-615-3p	6.72	8.46	9.5E-03	2.8E-03	**3.33**	**3.33**
	hsa-miR-324-3p	6.43	7.15	1.2E-02	7.0E-03	1.64	1.64
	hsa-miR-197-3p	1.94	3.13	2.6E-01	1.1E-01	**2.28**	**2.28**
	hsa-miR-296-3p	6.72	8.46	9.5E-03	2.8E-03	**3.33**	**3.33**
	hsa-miR-328	6.72	8.23	9.5E-03	3.3E-03	**2.84**	**2.84**
	hsa-miR-139-3p	6.25	8.46	1.3E-02	2.8E-03	**4.62**	**4.62**
	hsa-miR-339-5p	6.69	8.07	9.7E-03	3.7E-03	**2.60**	**2.60**
	hsa-miR-370	6.72	8.46	9.5E-03	2.8E-03	**3.33**	**3.33**
	hsa-miR-614	6.72	8.38	9.5E-03	3.0E-03	**3.15**	**3.15**
	hsa-miR-375	6.39	5.69	1.2E-02	1.9E-02	0.61	-1.63
	hsa-miR-330-3p	6.20	4.43	1.4E-02	4.6E-02	**0.29**	**-3.42**
	hsa-miR-193a-5p	3.61	3.74	8.2E-02	7.5E-02	1.09	1.09
	hsa-miR-498	6.72	8.46	9.5E-03	2.8E-03	**3.33**	**3.33**
	hsa-miR-346	6.72	7.42	9.5E-03	5.8E-03	1.62	1.62
	hsa-miR-663a	5.79	7.24	1.8E-02	6.6E-03	**2.73**	**2.73**
	hsa-miR-611	6.72	8.46	9.5E-03	2.8E-03	**3.33**	**3.33**
	hsa-miR-637	6.72	8.46	9.5E-03	2.8E-03	**3.33**	**3.33**
	hsa-miR-339-3p	4.90	6.17	3.3E-02	1.4E-02	**2.41**	**2.41**
	hsa-miR-323a-5p	6.72	8.36	9.5E-03	3.0E-03	**3.11**	**3.11**
	hsa-miR-661	5.14	7.00	2.8E-02	7.8E-03	**3.62**	**3.62**
	hsa-miR-487b	6.70	7.70	9.6E-03	4.8E-03	2.00	2.00
	hsa-let-7g-5p	0.49	0.99	7.1E-01	5.0E-01	1.41	1.41
	hsa-miR-374a-5p	6.72	8.46	9.5E-03	2.8E-03	**3.33**	**3.33**
	hsa-miR-374b-5p	6.05	6.08	1.5E-02	1.5E-02	1.02	1.02
	hsa-let-7i-5p	-0.21	-0.22	1.2E+00	1.2E+00	0.99	-1.01
	hsa-let-7d-5p	-0.77	-0.88	1.7E+00	1.8E+00	0.92	-1.08
	hsa-miR-200a-3p	6.72	8.46	9.5E-03	2.8E-03	**3.33**	**3.33**
	hsa-miR-148a-3p	6.72	5.79	9.5E-03	1.8E-02	0.52	-1.91
	hsa-miR-376b	6.71	8.32	9.6E-03	3.1E-03	**3.05**	**3.05**
	hsa-miR-376a-3p	6.71	8.32	9.6E-03	3.1E-03	**3.05**	**3.05**
	hsa-miR-519a-3p	6.71	8.32	9.6E-03	3.1E-03	**3.05**	**3.05**
	hsa-miR-376c	6.71	8.32	9.6E-03	3.1E-03	**3.05**	**3.05**

	hsa-miR-98	4.21	4.06	5.4E-02	6.0E-02	0.90	-1.11
	hsa-miR-302b-3p	6.71	8.32	9.6E-03	3.1E-03	**3.05**	**3.05**
	hsa-miR-141-3p	6.71	8.32	9.6E-03	3.1E-03	**3.05**	**3.05**
	hsa-miR-20a-5p	3.03	3.00	1.2E-01	1.3E-01	0.98	-1.02
	hsa-miR-200b-3p	6.71	8.32	9.6E-03	3.1E-03	**3.05**	**3.05**
	hsa-miR-148b-3p	6.71	8.32	9.6E-03	3.1E-03	**3.05**	**3.05**
	hsa-miR-215	6.71	8.32	9.6E-03	3.1E-03	**3.05**	**3.05**
	hsa-miR-320d	6.42	7.80	1.2E-02	4.5E-03	**2.60**	**2.60**
	hsa-miR-519e-3p	6.71	8.32	9.6E-03	3.1E-03	**3.05**	**3.05**
	hsa-miR-302d-3p	6.71	8.32	9.6E-03	3.1E-03	**3.05**	**3.05**
	hsa-miR-449a	6.71	8.32	9.6E-03	3.1E-03	**3.05**	**3.05**
	hsa-miR-146a-5p	6.71	7.89	9.6E-03	4.2E-03	**2.26**	**2.26**
	hsa-miR-20b-5p	3.58	3.96	8.4E-02	6.4E-02	1.30	1.30
	hsa-miR-192-5p	4.46	6.17	4.5E-02	1.4E-02	**3.26**	**3.26**
	hsa-miR-301a-3p	5.24	3.42	2.6E-02	9.4E-02	**0.28**	**-3.54**
	hsa-miR-320c	6.71	8.13	9.6E-03	3.6E-03	**2.67**	**2.67**
	hsa-miR-520a-3p	6.71	8.32	9.6E-03	3.1E-03	**3.05**	**3.05**
	hsa-miR-519d	6.71	8.32	9.6E-03	3.1E-03	**3.05**	**3.05**
	hsa-miR-204-5p	6.71	8.32	9.6E-03	3.1E-03	**3.05**	**3.05**
	hsa-miR-449b-5p	6.71	8.32	9.6E-03	3.1E-03	**3.05**	**3.05**
**Plate 4**	hsa-miR-301b	4.45	5.26	4.6E-02	2.6E-02	1.75	1.75
	hsa-miR-199a-5p	5.61	7.22	2.0E-02	6.7E-03	**3.05**	**3.05**
	hsa-miR-130b-3p	6.71	8.32	9.6E-03	3.1E-03	**3.05**	**3.05**
	hsa-miR-200c-3p	6.16	5.27	1.4E-02	2.6E-02	0.54	-1.86
	hsa-miR-302c-3p	6.71	8.32	9.6E-03	3.1E-03	**3.05**	**3.05**
	hsa-miR-211-5p	6.71	8.32	9.6E-03	3.1E-03	**3.05**	**3.05**
	hsa-miR-501-3p	6.71	8.32	9.6E-03	3.1E-03	**3.05**	**3.05**
	hsa-miR-10a-5p	4.77	4.63	3.7E-02	4.1E-02	0.91	-1.10
	hsa-miR-10b-5p	6.71	8.32	9.6E-03	3.1E-03	**3.05**	**3.05**
	hsa-let-7c	-0.94	-1.14	1.9E+00	2.2E+00	0.87	-1.14
	hsa-let-7b-5p	1.42	1.04	3.7E-01	4.9E-01	0.77	-1.30

	hsa-miR-135a-5p	6.71	8.32	9.6E-03	3.1E-03	**3.05**	**3.05**
	hsa-miR-18a-5p	6.71	8.32	9.6E-03	3.1E-03	**3.05**	**3.05**
	hsa-miR-196a-5p	6.71	6.81	9.6E-03	8.9E-03	1.07	1.07
	hsa-miR-18b-5p	6.71	8.32	9.6E-03	3.1E-03	**3.05**	**3.05**
	hsa-miR-520c-3p	6.71	8.32	9.6E-03	3.1E-03	**3.05**	**3.05**
	hsa-miR-135b-5p	6.71	8.32	9.6E-03	3.1E-03	**3.05**	**3.05**
	hsa-miR-106a-5p	3.05	2.74	1.2E-01	1.5E-01	0.80	-1.24
	hsa-miR-517a-3p	6.71	8.32	9.6E-03	3.1E-03	**3.05**	**3.05**
	hsa-miR-181c-5p	6.71	8.32	9.6E-03	3.1E-03	**3.05**	**3.05**
	hsa-miR-17-5p	3.30	2.92	1.0E-01	1.3E-01	0.77	-1.30
	hsa-miR-29a-3p	1.50	0.80	3.5E-01	5.8E-01	0.61	-1.63
	hsa-miR-29c-3p	6.71	8.32	9.6E-03	3.1E-03	**3.05**	**3.05**
	hsa-miR-196b-5p	6.71	7.66	9.6E-03	5.0E-03	1.93	1.93
	hsa-miR-520e	6.56	8.32	1.1E-02	3.1E-03	**3.38**	**3.38**
	hsa-miR-27b-3p	2.60	2.33	1.7E-01	2.0E-01	0.83	-1.21
	hsa-miR-19a-3p	6.71	8.32	9.6E-03	3.1E-03	**3.05**	**3.05**
	hsa-miR-27a-3p	6.71	5.41	9.6E-03	2.4E-02	0.41	**-2.47**
	hsa-miR-181a-5p	6.71	6.83	9.6E-03	8.8E-03	1.08	1.08
	hsa-miR-103a-3p	3.41	2.60	9.4E-02	1.7E-01	0.57	-1.76
	hsa-miR-107	3.25	4.95	1.1E-01	3.2E-02	**3.24**	**3.24**
	hsa-miR-133b	6.71	8.32	9.6E-03	3.1E-03	**3.05**	**3.05**
	hsa-miR-19b-3p	5.54	5.38	2.2E-02	2.4E-02	0.89	-1.12
	hsa-miR-181b-5p	4.90	5.23	3.4E-02	2.7E-02	1.25	1.25
	hsa-miR-23a-3p	-0.03	-0.18	1.0E+00	1.1E+00	0.90	-1.11
	hsa-miR-320a	-1.75	0.34	3.4E+00	7.9E-01	**4.25**	**4.25**
	hsa-miR-320b	2.25	3.80	2.1E-01	7.2E-02	**2.92**	**2.92**
	hsa-miR-133a	6.71	8.32	9.6E-03	3.1E-03	**3.05**	**3.05**
	hsa-miR-449c-5p	1.44	2.80	3.7E-01	1.4E-01	**2.56**	**2.56**
	hsa-miR-199a-3p	6.71	8.32	9.6E-03	3.1E-03	**3.05**	**3.05**
	hsa-miR-520c-5p	6.71	8.32	9.6E-03	3.1E-03	**3.05**	**3.05**
	hsa-miR-374a-3p	6.71	7.91	9.6E-03	4.2E-03	**2.29**	**2.29**
	hsa-miR-181c-3p	6.71	8.32	9.6E-03	3.1E-03	**3.05**	**3.05**

	hsa-miR-154-3p	6.71	8.18	9.6E-03	3.5E-03	**2.76**	**2.76**
	hsa-miR-377-5p	6.71	8.32	9.6E-03	3.1E-03	**3.05**	**3.05**
	hsa-miR-432-3p	6.71	8.32	9.6E-03	3.1E-03	**3.05**	**3.05**
	hsa-miR-181a-3p	6.71	8.32	9.6E-03	3.1E-03	**3.05**	**3.05**
	hsa-miR-192-3p	6.71	8.32	9.6E-03	3.1E-03	**3.05**	**3.05**
	hsa-miR-181a-2-3p	6.71	8.25	9.6E-03	3.3E-03	**2.90**	**2.90**
	hsa-miR-138-1-3p	4.59	5.04	4.2E-02	3.1E-02	1.36	1.36
	hsa-miR-34a-3p	6.71	8.32	9.6E-03	3.1E-03	**3.05**	**3.05**
	hsa-miR-135b-3p	6.71	8.32	9.6E-03	3.1E-03	**3.05**	**3.05**
	hsa-miR-221-5p	6.71	5.83	9.6E-03	1.8E-02	0.54	-1.84
	hsa-miR-200b-5p	6.71	8.32	9.6E-03	3.1E-03	**3.05**	**3.05**
	hsa-let-7a-3p	6.71	8.32	9.6E-03	3.1E-03	**3.05**	**3.05**
	hsa-let-7b-3p	5.54	7.05	2.2E-02	7.6E-03	**2.84**	**2.84**
	hsa-let-7c*	6.71	8.32	9.6E-03	3.1E-03	**3.05**	**3.05**
	hsa-let-7d-3p	4.55	6.03	4.3E-02	1.5E-02	**2.78**	**2.78**
	hsa-let-7e-3p	6.71	7.76	9.6E-03	4.6E-03	**2.07**	**2.07**
	hsa-let-7f-1-3p	6.71	7.94	9.6E-03	4.1E-03	**2.34**	**2.34**
	hsa-let-7f-2-3p	6.75	8.15	9.3E-03	3.5E-03	**2.64**	**2.64**
	hsa-let-7i-3p	6.75	8.15	9.3E-03	3.5E-03	**2.64**	**2.64**
	hsa-miR-100-3p	6.75	8.15	9.3E-03	3.5E-03	**2.64**	**2.64**
	hsa-miR-101-5p	6.75	8.15	9.3E-03	3.5E-03	**2.64**	**2.64**
	hsa-miR-106b-3p	6.22	7.00	1.3E-02	7.8E-03	1.72	1.72
	hsa-miR-10a-3p	6.75	8.15	9.3E-03	3.5E-03	**2.64**	**2.64**
	hsa-miR-10b-3p	6.75	8.15	9.3E-03	3.5E-03	**2.64**	**2.64**
	hsa-miR-122-3p	6.75	8.15	9.3E-03	3.5E-03	**2.64**	**2.64**
	hsa-miR-124-5p	6.75	8.15	9.3E-03	3.5E-03	**2.64**	**2.64**
	hsa-miR-129-1-3p	6.75	7.56	9.3E-03	5.3E-03	1.75	1.75
	hsa-miR-130b-5p	6.75	8.15	9.3E-03	3.5E-03	**2.64**	**2.64**
	hsa-miR-132-5p	6.75	8.15	9.3E-03	3.5E-03	**2.64**	**2.64**
	hsa-miR-135a-3p	6.75	8.15	9.3E-03	3.5E-03	**2.64**	**2.64**
	hsa-miR-138-2-3p	6.75	8.15	9.3E-03	3.5E-03	**2.64**	**2.64**
	hsa-miR-143-5p	6.75	8.15	9.3E-03	3.5E-03	**2.64**	**2.64**
	hsa-miR-144-5p	6.75	8.15	9.3E-03	3.5E-03	**2.64**	**2.64**

	hsa-miR-145-3p	6.75	8.15	9.3E-03	3.5E-03	**2.64**	**2.64**
	hsa-miR-146a-3p	6.75	8.15	9.3E-03	3.5E-03	**2.64**	**2.64**
	hsa-miR-148a-5p	6.75	8.15	9.3E-03	3.5E-03	**2.64**	**2.64**
	hsa-miR-155-3p	3.66	0.87	7.9E-02	5.5E-01	**0.14**	**-6.92**
	hsa-miR-186-3p	6.75	8.15	9.3E-03	3.5E-03	**2.64**	**2.64**
	hsa-miR-200c-5p	6.65	8.05	1.0E-02	3.8E-03	**2.64**	**2.64**
	hsa-miR-202-5p	6.75	8.15	9.3E-03	3.5E-03	**2.64**	**2.64**
	hsa-miR-20a-3p	6.75	8.15	9.3E-03	3.5E-03	**2.64**	**2.64**
	hsa-miR-214-5p	6.75	8.15	9.3E-03	3.5E-03	**2.64**	**2.64**
	hsa-miR-218-2-3p	6.75	7.82	9.3E-03	4.4E-03	**2.10**	**2.10**
	hsa-miR-22-5p	6.75	7.82	9.3E-03	4.4E-03	**2.10**	**2.10**
	hsa-miR-223-5p	6.75	8.15	9.3E-03	3.5E-03	**2.64**	**2.64**
	hsa-miR-23a-5p	6.75	8.05	9.3E-03	3.8E-03	**2.46**	**2.46**
	hsa-miR-23b-5p	6.75	8.15	9.3E-03	3.5E-03	**2.64**	**2.64**
	hsa-miR-24-1-5p	6.75	8.15	9.3E-03	3.5E-03	**2.64**	**2.64**
	hsa-miR-24-2-5p	6.75	8.15	9.3E-03	3.5E-03	**2.64**	**2.64**
**Plate 5**	hsa-miR-25-5p	6.75	8.15	9.3E-03	3.5E-03	**2.64**	**2.64**
	hsa-miR-26a-1-3p	6.75	8.15	9.3E-03	3.5E-03	**2.64**	**2.64**
	hsa-miR-26a-2-3p	6.75	8.15	9.3E-03	3.5E-03	**2.64**	**2.64**
	hsa-miR-27b-5p	6.67	8.15	9.8E-03	3.5E-03	**2.79**	**2.79**
	hsa-miR-29a-5p	6.75	8.06	9.3E-03	3.8E-03	**2.48**	**2.48**
	hsa-miR-29b-1-5p	5.24	4.12	2.6E-02	5.8E-02	0.46	**-2.17**
	hsa-miR-29b-2-5p	6.75	8.15	9.3E-03	3.5E-03	**2.64**	**2.64**
	hsa-miR-29c-5p	6.75	8.15	9.3E-03	3.5E-03	**2.64**	**2.64**
	hsa-miR-30c-1-3p	5.91	8.15	1.7E-02	3.5E-03	**4.72**	**4.72**
	hsa-miR-30d-3p	5.70	6.15	1.9E-02	1.4E-02	1.37	1.37
	hsa-miR-31-3p	6.75	8.10	9.3E-03	3.6E-03	**2.55**	**2.55**
	hsa-miR-32-3p	6.75	8.15	9.3E-03	3.5E-03	**2.64**	**2.64**
	hsa-miR-335-3p	6.75	8.15	9.3E-03	3.5E-03	**2.64**	**2.64**
	hsa-miR-33a-3p	4.62	5.37	4.1E-02	2.4E-02	1.68	1.68
	hsa-miR-340-3p	6.75	8.15	9.3E-03	3.5E-03	**2.64**	**2.64**
	hsa-miR-34b-5p	6.75	8.15	9.3E-03	3.5E-03	**2.64**	**2.64**
	hsa-miR-367-5p	6.75	8.15	9.3E-03	3.5E-03	**2.64**	**2.64**
	hsa-miR-374b-3p	6.75	8.15	9.3E-03	3.5E-03	**2.64**	**2.64**

	hsa-miR-378a-5p	6.75	8.15	9.3E-03	3.5E-03	**2.64**	**2.64**
	hsa-miR-424-3p	6.34	7.26	1.2E-02	6.5E-03	1.89	1.89
	hsa-miR-497-3p	6.75	8.15	9.3E-03	3.5E-03	**2.64**	**2.64**
	hsa-miR-500a-3p	6.75	8.15	9.3E-03	3.5E-03	**2.64**	**2.64**
	hsa-miR-517-5p	6.75	8.15	9.3E-03	3.5E-03	**2.64**	**2.64**
	hsa-miR-708-3p	6.75	8.15	9.3E-03	3.5E-03	**2.64**	**2.64**
	hsa-miR-7-1-3p	5.37	7.38	2.4E-02	6.0E-03	**4.03**	**4.03**
	hsa-miR-7-2-3p	6.75	8.15	9.3E-03	3.5E-03	**2.64**	**2.64**
	hsa-miR-9-3p	6.75	8.15	9.3E-03	3.5E-03	**2.64**	**2.64**
	hsa-miR-93-3p	6.75	8.15	9.3E-03	3.5E-03	**2.64**	**2.64**
	hsa-miR-99a-3p	6.75	8.15	9.3E-03	3.5E-03	**2.64**	**2.64**
	hsa-miR-99b-3p	6.75	8.15	9.3E-03	3.5E-03	**2.64**	**2.64**
	hsa-miR-224-3p	2.28	3.44	2.1E-01	9.2E-02	**2.23**	**2.23**
	hsa-miR-103a-2-5p	6.75	8.15	9.3E-03	3.5E-03	**2.64**	**2.64**
	hsa-miR-449b-3p	5.80	6.22	1.8E-02	1.3E-02	1.34	1.34
	hsa-let-7a-2-3p	6.75	7.31	9.3E-03	6.3E-03	1.47	1.47
	hsa-miR-320e	4.38	7.01	4.8E-02	7.8E-03	**6.19**	**6.19**
	hsa-miR-125b-1-3p	6.75	8.15	9.3E-03	3.5E-03	**2.64**	**2.64**
	hsa-miR-125b-2-3p	4.53	3.59	4.3E-02	8.3E-02	0.52	-1.92
	hsa-miR-130a-5p	5.87	7.56	1.7E-02	5.3E-03	**3.23**	**3.23**
	hsa-miR-141-5p	6.75	8.15	9.3E-03	3.5E-03	**2.64**	**2.64**
	hsa-miR-148b-5p	5.22	8.15	2.7E-02	3.5E-03	**7.62**	**7.62**
	hsa-miR-149-3p	5.40	6.11	2.4E-02	1.4E-02	1.64	1.64
	hsa-miR-150-3p	2.70	3.83	1.5E-01	7.0E-02	**2.19**	**2.19**
	hsa-miR-15a-3p	4.99	5.88	3.2E-02	1.7E-02	1.85	1.85
	hsa-miR-15b-3p	3.95	6.65	6.5E-02	1.0E-02	**6.50**	**6.50**
	hsa-miR-16-1-3p	6.75	8.15	9.3E-03	3.5E-03	**2.64**	**2.64**
	hsa-miR-16-2-3p	6.75	7.05	9.3E-03	7.6E-03	1.23	1.23
	hsa-miR-17-3p	4.36	7.94	4.9E-02	4.1E-03	**11.96**	**11.96**
	hsa-miR-182-3p	6.71	8.15	9.6E-03	3.5E-03	**2.71**	**2.71**
	hsa-miR-183-3p	6.75	8.15	9.3E-03	3.5E-03	**2.64**	**2.64**
	hsa-miR-18b-3p	6.23	8.15	1.3E-02	3.5E-03	**3.78**	**3.78**
	hsa-miR-191-3p	6.56	8.15	1.1E-02	3.5E-03	**3.01**	**3.01**
	hsa-miR-193b-5p	4.75	6.04	3.7E-02	1.5E-02	**2.45**	**2.45**
