# Supplementary material for: Dysregulation of the miR‐194–CUL4B negative feedback loop drives tumorigenesis in non‐small‐cell lung carcinoma
Source: Mol Oncol. 2017 Feb 21;11(3):305–19. doi: 10.1002/1878-0261.12038 (PMC5527444; doi:10.1002/1878-0261.12038)
Supplement: Supplementary file 2 — Fig. S1. Knockdown of CUL4B inhibits proliferation, migration and invasion of NSCLC cells. Fig. S2. CUL4B is a target of miR‐194. Fig. S3. p53 downregulates CUL4B by transactivating miR‐194. Fig. S4. CUL4B represses miR‐194 expression. [file MOL2-11-305-s002.docx]

**Supplementary Figures and figure legends**

**Supplementary Figure 1**


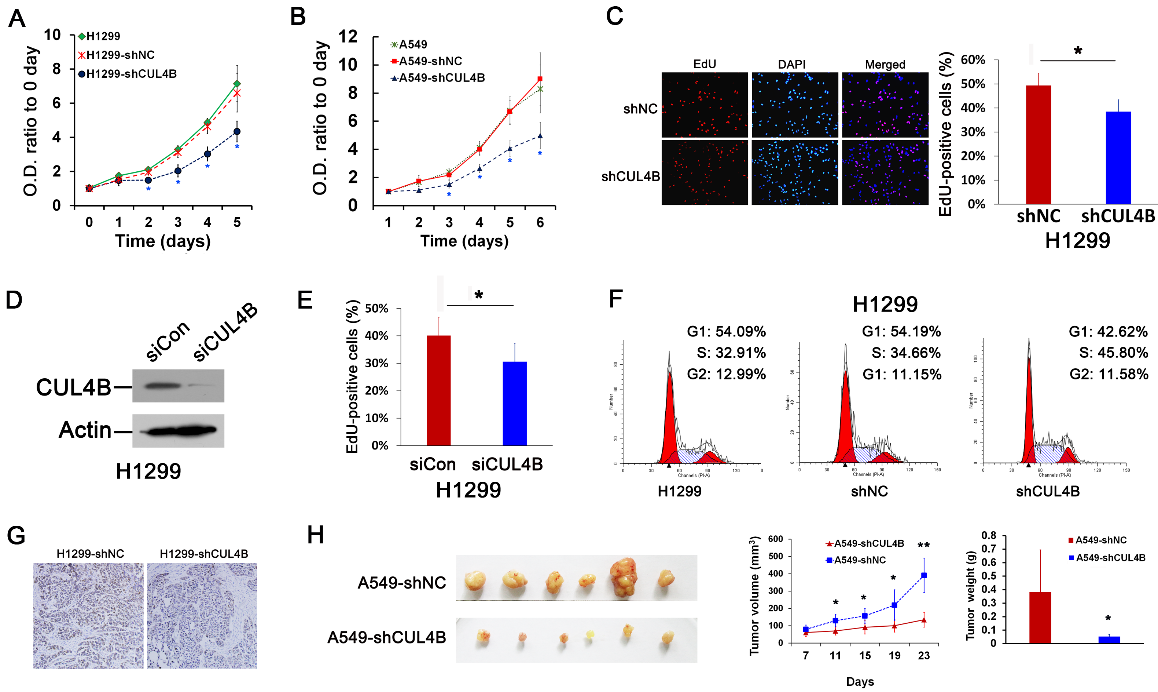


Supplementary Figure 1. Knockdown of CUL4B inhibits proliferation, migration and invasion of NSCLC cells. (A, B) MTT assays of wildtype, CUL4B knockdown and control H1299 (A) and A549 (B) cells at different time points. The data in each time point are the mean results ± S.D. of the averaged values from 8 replicates. *, p<0.001. (C) EdU incorporation assay of shCUL4B and shNC H1299 cells. Error bars represent the S.D. **P* < 0.05. (D, E) H1299 cells were transiently transfected with siCUL4B and control siRNA. 72 h later, CUL4B expression was detected by Western blot analysis (D) and DNA replication was measured by EdU incorporation (E), Error bars represent the S.D. **P* < 0.05. (F) H1299 cells were transiently transfected with siCUL4B and control siRNA. 72 h later, cell cycle distribution was determined by flow cytometric analysis. (G) Expression of Ki67 in the H1299 xenograft tissues was examined by IHC. (H) shCUL4B and control A549 cells were injected into nude mice, respectively. The tumor volume was monitored and recorded. Photographs illustrate representative tumors 23 days after injection and the tumor weight was measured. Error bars represent the S.D. *, p<0.05.

**Supplementary Figure 2**


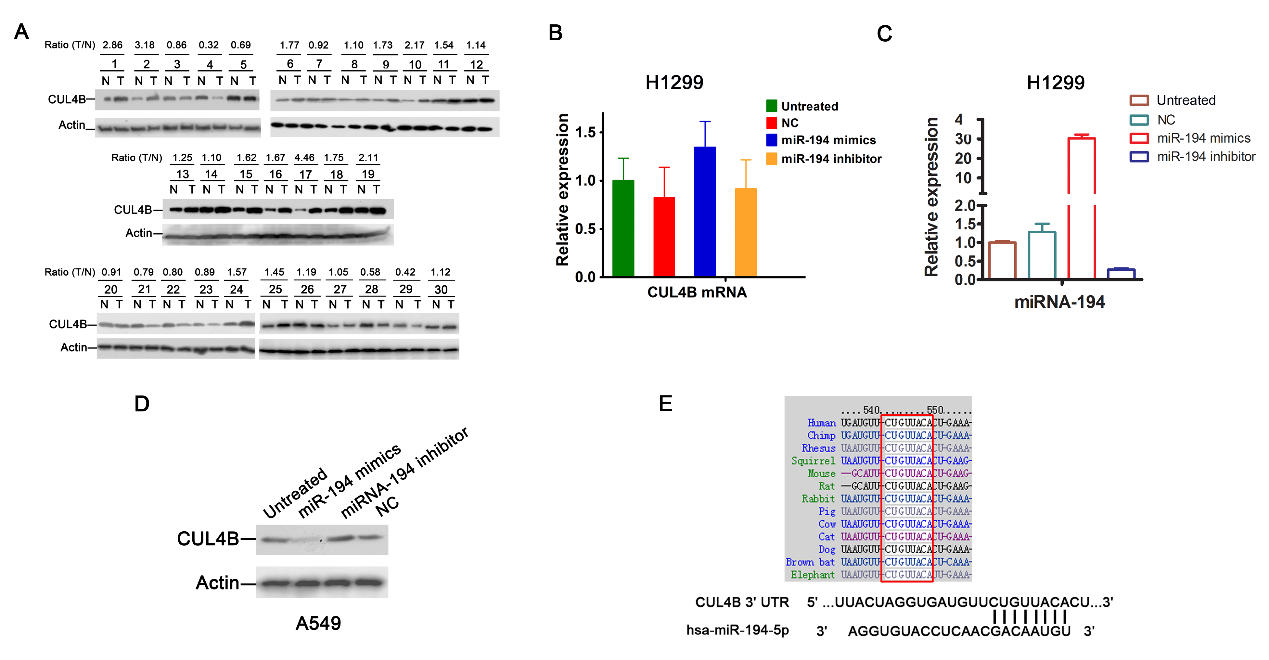


Supplementary Figure 2. CUL4B is a target of miR-194. **(**A) Western blot analysis of CUL4B protein levels in lung cancer tissues (T) and the paired adjacent non-tumor tissues (N). Proteins levels were quantified by densitometric analysis using ImageJ. (B， C) H1299 cells were transiently transfected with miR-194 mimics, inhibitor or negative control RNA (NC), respectively. 72 h later, CUL4B (B) and miR-194 (C) levels were determined by real-time PCR. Error bars represent the SEM. (D) A549 cells were transiently transfected with miR-194 mimics, inhibitor or negative control RNA (NC). 72 h later, CUL4B protein levels were determined by Western blot. (E) Alignments of the human CUL4B 3’ UTR with orthologous sequences. The miR-194 targeting site is boxed, and nucleotides corresponding to the human sequence are also shown.

**Supplementary Figure 3**

**
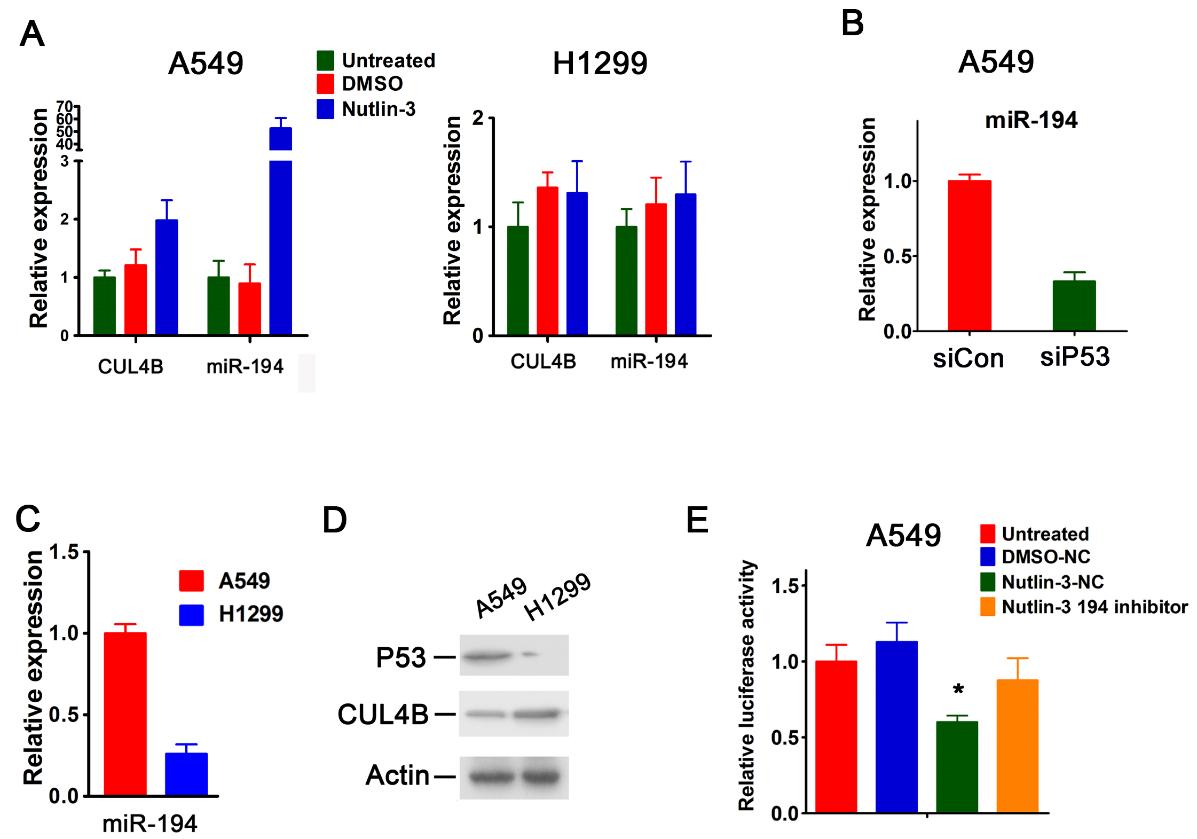
**

Supplementary Figure 3. p53 downregulates CUL4B by transactivating miR-194. **(**A) A549 cells and H1299 cells were treated with or without nutlin-3. 48 h later, expression of CUL4B and miR-194 were examined by real-time PCR, Bars represent SEM. (B) A549 cells were transiently transfected with p53 siRNA or control siRNA. 72 h later, miR-194 levels were determined by real-time PCR, Bars represent SEM. (C) miR-194 levels in H1299 and A549 cells were determined by real-time PCR, Bars represent SEM. (D) CUL4B levels in H1299 and A549 cells were determined by Western blot. (E) A549 cells cotransfected with miR-194 inhibitor or control RNA and wildtype pmir-GLO-CUL4B-3’ UTR vector were treated with or without nutlin-3 and luciferase activity were measured. Error bars represent the S.D. *, p<0.05 versus DMSO treated NC transfected cells.

**Supplementary Figure 4**


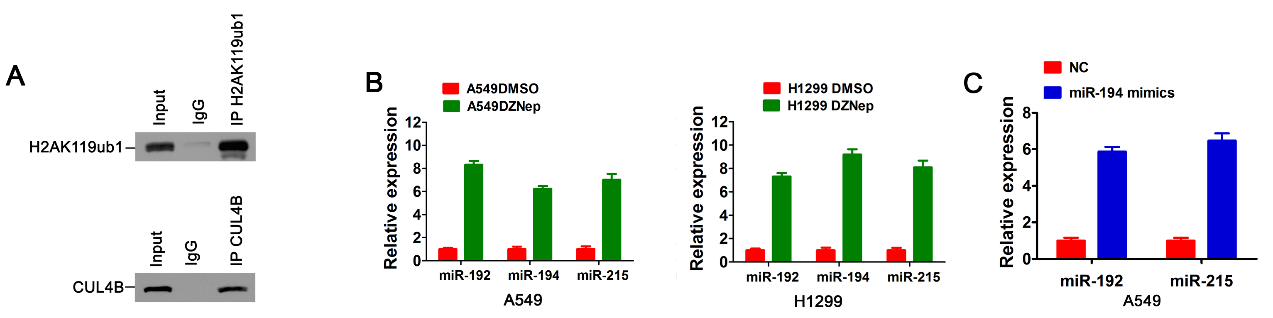


Supplementary Figure 4. CUL4B represses miR-194 expression. **(**A) ChIP experiments were performed using anti-H2AK119ub1 or anti-CUL4B antibody and the pulldown proteins were analyzed by western blot. **(**B) A549 and H1299 cells were treated with 10μM DZNep or DMSO for 36 h and expression of indicated miRNAs was determined using real-time PCR. The levels in DMSO treated cells were set as 1. Bars represent SEM. (C) H1299 cells were transfected with miR-194 mimics or control RNA. 48 h later, expression of miR-192 and -215 was determined using real-time PCR. Error bars represent the SEM.
