# Supplementary material for: Dysregulation of the miR‐194–CUL4B negative feedback loop drives tumorigenesis in non‐small‐cell lung carcinoma
Source: Mol Oncol. 2017 Feb 21;11(3):305–19. doi: 10.1002/1878-0261.12038 (PMC5527444; doi:10.1002/1878-0261.12038)
Supplement: Supplementary file 3 — Table S1. Primer sequences used for qPCR. Table S2. Primary antibodies for western blot. Table S3. Primer sequences used for site‐directed mutagenesis. Table S4. Primer sequences used for ChIP‐qPCR. Table S5. Primary antibodies for ChIP. Table S6. Correlation of the High CUL4B Expression with Clinicopathological Characteristics. [file MOL2-11-305-s003.pdf]

Supplementary Tables

Supplementary Table 1: Primer sequences used for qPCR

Primer name	Primer sequence (5'to 3')
CUL4B-F	TGCTGCTCAGGAGGTCAGATC
CUL4B-R	TGGAATCAAAGTCTTCTCTCTCGTT
GAPDH-F	ACAACAGCCTCAAGATCATCAG
GAPDH-F	GGTCCACCACTGACACGTTG
TIMP-3-F	CAGGTCGCGTCTATGATGGC
TIMP-3-R	AGGTGATACCGATAGTTCAGCC
N-cadherin-F	AGCCAACCTTAACTGAGGAGT
N-cadherin-R	GGCAAGTTGATTGGAGGGATG
Vimentin-F	GACGCCATCAACACCGAGTT
Vimentin-R	CTTTGTCGTTGGTTAGCTGGT
TWIST1-F	GTCCGCAGTCTTACGAGGAG
TWIST1-R	GCTTGAGGGTCTGAATCTTGCT
pri-miR-194-1-F	AGCGTTTCAAATCTACCAGT
pri-miR-194-1-R	TATCTTCTGTGTACCTGCCA
pri-miR-194-2-F	ATGATAAGAAGCCTCGGTGA
pri-miR-194-2-R	GTGGGACCATGAGTGCTGCA

Supplementary Table 2: Primary antibodies for Western blot

Name	manufacturer and catalog numbers
mouse anti-β-actin	Santa Cruz Biotechnology, Inc.sc-69879
mouse anti-p53	Abcam, ab28
mouse anti- N-cadherin	Proteintech, 66219-1-Ig
rabbit anti-MDM2	Proteintech, 19058-1-AP
rabbit anti-RBX1	Proteintech, 14895-1-AP
rabbit anti-CUL4B	Sigma-Aldrich, C9995

Supplementary Table 3: Primer sequences used for site-directed mutagenesis

Primer name	Primer sequence (5'to 3')
pmir-GLO-CUL4B 3'UTR-WT-F	GAGCTCAGATCAATCAAATCCAGATGAA
pmir-GLO-CUL4B 3'UTR-WT-R	CCGCTCGAGCAGTGACAGTAAAGAGAAGCCT
pmir-GLO-CUL4B 3'UTR-MU-F	CTAGGTGATGTTCTCAATGACTGAAAGGGAT
pmir-GLO-CUL4B 3'UTR-MU-R	ATCCCTTTCAGTCATTGAGAACATCACCTAG
pmir-GLO-RBX1 3'UTR-WT-F	GAGCTCGAAAAGACTTCTTCCATC
pmir-GLO-RBX1 3'UTR-WT-R	CCGCTCGAGGAAAGTTCAGAGTAGAGA
pmir-GLO-RBX1 3'UTR-MU-F	TGACTTTCCCTGCTCAATCCTAATTAC
pmir-GLO-RBX1 3'UTR-MU-R	GTAATTAGGATTGAGCAGGGAAAGTCA
pmir-GLO- N-cadherin 3'UTR-WT-F	GAGCTCGGGAGCAGTAAGTTAAACCA
pmir-GLO- N-cadherin 3'UTR-WT-R	CCGCTCGAGGTAGTGCCACCAGTGTCAGG
pmir-GLO- N-cadherin 3'UTR-MU-F	GCTTCAACACGCTTTAGTAACTTTGCATTTGCTTTTA
pmir-GLO- N-cadherin 3'UTR-MU-R	TAAAAGCAAATGCAAAGTTACTAAAGCGTGTTGAAGC

Supplementary Table 4: Primer sequences used for ChIP-qPCR

Primer name	Primer sequence (5'to 3')
194-192-1-F	AGGTCCCTGGGGACTGACAAG
194-192-1-R	GAGCGGCTCGGATTTACAACT
194-192-2-F	CTTTGTCACTGAGGCTGGAGT
194-192-2-R	AGAAGAGTAAGGCCGAGGATG
194-192-3-F	CTGTCTGGGAGGGACGAGAAC
194-192-3-R	GGTGACCTTGAGCAAGTAGCG
194-192-4-F	CCTGCTCATGGCATGTCTTGT
194-192-4-R	CCTGCTCATGGCATGTCTTGT
194-192-5-F	TGGGAATAAGAGGAGGAGGTG
194-192-5-R	GCTGTGGATGAGCCAAAGGAG
194-192-6-F	AGCACGTCACGATGGACCAGG
194-192-6-R	CGATGAGGAGGCCAGCAAAAG
194-192-7-F	GGTCTACGTGAGGGCAGAGCC
194-192-7-R	AGGTCACAGACTGGGGATGGG
194-215-1-F	TAGTAACAGATTTCAACCCAA
194-215-1-R	GAAGAAAGTCTCGATTTATTCA
194-215-2-F	TTAAAGCTGACAGAAAACCAT
194-215-2-R	TGGGAAGGAAAAGAAGTTGGT
194-215-3-F	CAACTATGACATCAGCCAATC
194-215-3-R	TGAAACATTTAGCCTCTTACC
194-215-4-F	CTGGCAAGTTTAGGTAAAGAG
194-215-4-R	TCCTAACATTTCTACCGTATT
194-215-5-F	AGAGGGAGACTCCGTCTCAAA
194-215-5-R	CCCATTTTCCTTTCTCATTAT
194-215-6-F	GTTTCCGTTACTGATTTACCT
194-215-6-R	GACTGATACAGTGTCAGTGGG
194-215-7-F	GTTTCCGTTACTGATTTACCT
194-215-7-R	GACTGATACAGTGTCAGTGGG
194-215-8-F	GCCCACCATCGCACCCAGCTAAT
194-215-8-R	GAGGCGGGCAGATCACAAGGTCA

Supplementary Table 5: Primary antibodies for ChIP

Name	manufacturer and catalog numbers
rabbit anti-CUL4B	Sigma-Aldrich, C9995
rabbit anti-H2A	Abcam, ab177308
rabbit anti-H2AK119ub1	cell signaling, 8240P
rabbit anti-H3K4me3	Millipore, 07-473
mouse anti-H3K27me3	Abcam, ab6002
mouse anti-EZH2	BD biosciences, 612667

Supplementary Table 6: Correlation of the High CUL4B Expression with Clinicopathological Characteristics

Clinicopathologi characteristics	No. of the patients	High Expression, No. of Patients	χ2	*P*
**Gender**				
Male	39	20(0.51)	0.255	0.613
Female	35	20(0.57)		
**Age(years)**				
<=60	37	20(0.54)	0.05	0.824
>60	35	18(0.51)		
unknown	2	2(1)		
**Tumor size(mm)**				
<=30	28	20(0.71)	5.475	**0.019***
>30	46	20(0.43)		
**pathological classification**				
I	12	7(0.58)	0.437	0.804
II	49	27(0.56)		
III	13	6(0.46)		
**TNM Staging**				
I	26	10(0.59)	3.442	0.328
II	13	17(0.66)		
III	18	5(0.38)		
unknown	17	8(0.44)		
**Tumor status**				
T1	21	16(0.76)	5.801	0.055
T2	38	17(0.44)		
T3	15	7(0.46)		
**Lymph node metastasis**				
No	35	21(0.60)	1.551	0.213
Yes	21	9(0.43)		
unknown	15	8(0.53)		
**Three-year survival**				
yes	19	8(0.42)	1.47	0.225
no	55	32(0.58)		

* *P* <0 .05.
